# Reversible redox chemistry in azobenzene-based organic molecules for high-capacity and long-life nonaqueous redox flow batteries

**DOI:** 10.1038/s41467-020-17662-y

**Published:** 2020-07-31

**Authors:** Leyuan Zhang, Yumin Qian, Ruozhu Feng, Yu Ding, Xihong Zu, Changkun Zhang, Xuelin Guo, Wei Wang, Guihua Yu

**Affiliations:** 1 Materials Science and Engineering Program and Department of Mechanical Engineering, The University of Texas at Austin, Austin, TX 78712 USA; 2 Energy & Environment Directorate, Pacific Northwest National Laboratory, Richland, WA 99352 USA

**Keywords:** Energy storage, Materials for energy and catalysis

## Abstract

Redox-active organic molecules have drawn extensive interests in redox flow batteries (RFBs) as promising active materials, but employing them in nonaqueous systems is far limited in terms of useable capacity and cycling stability. Here we introduce azobenzene-based organic compounds as new active materials to realize high-performance nonaqueous RFBs with long cycling life and high capacity. It is capable to achieve a stable long cycling with a low capacity decay of 0.014% per cycle and 0.16% per day over 1000 cycles. The stable cycling under a high concentration of 1 M is also realized, delivering a high reversible capacity of ~46 Ah L^−1^. The unique lithium-coupled redox chemistry accompanied with a voltage increase is observed and revealed by experimental characterization and theoretical simulation. With the reversible redox activity of azo group in π-conjugated structures, azobenzene-based molecules represent a class of promising redox-active organics for potential grid-scale energy storage systems.

## Abstract (web summary)

Organic molecules are promising active materials for nonaqueous redox-flow batteries (RFBs), but suffer from poor cycling stability. Here, the authors introduce azobenzene-based molecules as new type of highly soluble and stable active materials to realize high-capacity and long-life nonaqueous RFBs.

## Introduction

Developing efficient energy storage technologies is essential to effectively alleviate the growing energy crisis and environmental issues^1^. Compared with the well-known lithium-ion batteries, redox flow batteries (RFBs) have been widely considered as a promising stationary energy storage candidate to utilize renewable resources, such as solar or wind power, due to their advantageous features of decoupled energy and power and design flexibility^1,2^. Ever since the initiation of RFB concept in 1960s, the developed battery systems can be often categorized into two types including aqueous and nonaqueous systems based on applied electrolytes.

Currently aqueous RFBs (ARFBs) have achieved tremendous progress from the viewpoint of successfully commercialized vanadium-based RFBs^3^. Since aqueous systems use redox-active materials dissolved in water as reactive electrolytes (anolyte and catholyte), they offer several attractive characteristics: nonflammability for safety benefits, high conductivity and fast reaction kinetics for high power operation, and potentially low cost for large-scale applications^4^. In addition, many selective ion-conductive membranes are available to support the design of high-performance ARFBs^5^. However, traditional ARFBs still suffer from the use of costly transition metal species, high electrolyte corrosivity and limited voltage and energy density^6^. In contrast, nonaqueous RFBs (NARFBs) have a great potential to realize the high voltage and energy density, but still face a number of scientific and technical challenges for practical implementation^7^. Specifically, despite the lack of appropriate selective ion-conductive membranes compatible with organic solvents, the key challenge lies in the choices of highly soluble and chemically stable redox species in organic supporting electrolytes to achieve a stable long cycling.

In recent years, organic active materials have rapidly emerged as a promising alternative to replace traditional metal-based materials for the development of next-generation RFBs^8–10^. Benefited from chemical diversity, structural tunability, elemental sustainability, and cost-effectiveness, the adoption of organic molecules in aqueous systems is successful as to improving solubility, tailoring redox potential and enhancing both chemical and electrochemical stability. Nevertheless, even with molecular engineering, the development of active organic molecules for NARFBs falls far behind the state-of-the-art ARFBs considering the limited current capability and serious performance decay^7^. Moreover, although high solubility (≥1 M) of several potential organic molecules, such as 9-fluorenone^11^, 2,1,3-benzothiadiazole^12^, 2,5-di-tert-butyl-1-methoxy-4-[2′-methoxyethoxy]benzene (DBMMB)^13^, 2,2,6,6-tetramethylpiperidine-1-oxyl^14^, and 1,1-dimethylferrocene (DMFc)^15^, have been reported in organic solvents, the cycling of assembled NARFBs was only demonstrated at low concentrations and it is still difficult to obtain a long-term cycling at high concentrations (1 M, >100 cycles, >500 h). Besides, a biredox electrolyte strategy^16^ was recently proposed to improve concentrations and accommodate cycling issues in nonaqueous systems; however, it can only apply to several specific molecules, and as the intrinsic instability of molecules still remains, the demonstrated cycling stability at high concentrations is limited. Thus, to accelerate the development of nonaqueous organic RFBs, searching new-type organic molecules with high solubility and excellent electrochemical stability is highly desired. In this work, a new design of molecular structure with intrinsically good stability is provided and the stable cycling at both low and high concentrations is achieved, presenting new insight into designing stable molecules for NARFBs.

Inspired by the realization of enhanced electrochemical stability by adopting extended π-conjugated structure^17–19^ and high solubility of aromatic hydrocarbons^20,21^, here we report a class of azo compounds as promising organic molecules for high-energy and stable NARFBs. In this study, three azo compounds, azobenzene (AB), 4-methoxyazobenzene (MAB), and 4-hydroxyazobenzene (HAB) that are composed of two phenyl rings with an azo functional group in the center, forming the π-conjugated structure, are explored as organic active materials to study the redox chemistry in organic solvents and electrochemical performance in NARFBs. As the basic compound, the AB molecule is mainly employed as the model material to investigate the electrochemical properties in different supporting electrolytes and it shows a high solubility (4–5 M) in common organic solvents, which is beneficial for achieving high energy density. Particularly, the polarization of AB induced by intermolecular interactions improves its solubility in the polar aprotic solvent, dimethylformamide (DMF). Since the electrochemical properties of AB are highly dependent on supporting electrolytes, the electrochemical performance is evaluated by using the optimal electrolyte design composed of AB molecules, lithium bis(trifluoromethanesulfonyl)imide (LiTFSI/LT) salts and DMF solvents. As a result, the demonstrated NARFB shows a reversible capacity as high as 46 Ah L^−1^ with a cell voltage of over 2 V. More importantly, it is capable of achieving stable cycling over 1000 h at both low (0.1 M) and high (1 M) concentrations with the capacity fade of only 0.007% per cycle/0.35% per day and 0.15% per cycle/0.16% per day, respectively. And given diverse derivatives available in industry, the combination of high solubility and excellent electrochemical stability of AB represents an important step forward towards the development of advanced NARFBs.

## Results

### Physical and electrochemical properties in organic solvents

Although several bio-inspired redox cofactors, such as quinone-, flavin-, alloxazine-, or phenazine-based organic derivatives, have been proposed as promising candidates for high-performance ARFBs^22–25^, it is still rarely reported that organic molecules have high solubility and excellent stability for NARFBs with high capacity and long cycle life. On the other hand, four different redox-active organics, free-radical (i.e. C–O^.^), organosulfur (–S_n_–)^26^, carbonyl (C=O), and imine (C=N) compounds have been mainly explored as active materials in RFBs up to now^8,27^. As a new type of organic compounds for RFB applications, the base molecule in the family, AB, has an azo group (N=N) in the center for reversible redox activity, which is connected by two phenyl rings, forming an extended π-conjugated structure. Similar to the design of nitrogen-containing heteroaromatic molecules^25,28^, it is believed that the π-conjugated structure may also enable a stable electrochemical reversibility of AB. As commercialized dyes in industry^29^, the abundant AB derivatives available in market with various functional groups make them even more attractive for practical relevance. Note that AB and its derivatives are known for the property of photoisomerization and the *trans*-isomer is thermodynamically stable at ambient environment^30^.

**Fig. 1:**
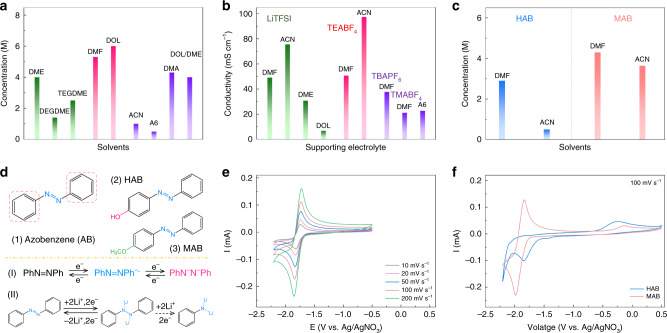
Physical properties and redox chemistry of AB derivatives. **a** Solubility measurement of AB in various organic solvents. **b** Ionic conductivities of various supporting electrolytes at a concentration of 0.5 M. **c** Solubility of two AB derivatives in DMF or ACN solvents. **d** Molecular structure of AB and its derivatives and possible reaction mechanisms for AB. CV curves of AB (**e**) and its derivatives (**f**) at a concentration of 10 mM in 0.5 M LiTFSI DMF supporting electrolytes.

We first conducted an extensive screening of supporting electrolytes including solvents and supporting salts to determine the optimal system with high solubility, high conductivity, appropriate redox potential, and good electrochemical reversibility. As the AB molecule has a planar configuration with a low polarity, it is hardly soluble in water. In contrast, the solubility measurement of AB (Fig. 1a) shows that it is highly soluble in various common organic solvents, which provides a great opportunity to maximize the energy density in NARFB design. Predictably, AB should show a high solubility in nonpolar solvents such as chloroform or diethyl carbonate (DEC) and a low solubility in polar solvents such as ethylene carbonate (EC), acetonitrile (ACN), or N,N-DMF. Thus, according to the difference of dielectric constant (Supplementary Fig. 1a), it can be understood that AB is highly soluble (≥1 M) in organic solvents with low dielectric constant, including 1,2-dimethoxyethane (DME), diglyme (DEGDME), tetraglyme (TEGDME), and 1,3-dioxolane (DOL). But it has a limited solubility in ACN, which is a common solvent for NARFBs, because of high dielectric constant. However, high solubility of AB (>4 M) is obtained in polar DMF or dimethylacetamide (DMA) solvents. According to the UV–vis spectra of AB in different solvents (Supplementary Fig. 1b–d), it is confirmed the *trans*-structure is stable in applied solvents, and additionally, the high-intensity peak in ultraviolet region corresponding to the π–π* absorption of phenyl rings has a clear red shift in DMF, implying the intermolecular interactions between AB and DMF. Given the resonance structure of DMF molecules, the polarization of AB caused by the induction force results in an improved solubility. As DMA has a similar molecular structure with DMF (Supplementary Fig. 1e), a high solubility of AB is also obtained. Except for solubility of active materials, conductivity of supporting electrolytes is another important factor that influences the performance of RFBs. Via impedance spectroscopy, the ionic conductivities of various supporting electrolytes are determined (Fig. 1b). When using common supporting salts, either LiTFSI or tetraethylammonium tetrafluoroborate (TEABF_4_), DMF, and ACN stand out as promising alternatives for supporting electrolytes with a high conductivity. It is found that TEABF_4_ is hardly soluble in DOL or DME solvents, and tetramethylammonium tetrafluoroborate (TMABF_4_) shows a low solubility in DMF, leading to the limited conductivity. Considering both solubility and conductivity factors, DMF is the optimal candidate for highly concentrated electrolyte design of AB. Further to explore the possibility of molecular functionalization on tuning solubility, redox potentials and reversibility of AB, two electron-donating groups, hydroxyl and methoxy, are chosen in the *para*-substituted position based on their different polarities (Fig. 1c, d). Intriguingly, these two derivatives maintain the high solubility of the parent AB in DMF while exhibit different dissolving behavior in ACN (Fig. 1c). It is worth mentioning that both polar and nonpolar groups substituted in the parent AB can increase the polarity because of inductive effect and increased asymmetry. However, the substituted hydroxy group should be likely to form hydrogen bonds with azo groups or adjacent hydroxy groups, leading to the decreased solubility in ACN. In DMF solvents, because carbonyl groups can also form hydrogen bond with hydroxy groups as the solvation interaction, a high solubility (~3 M) could still be retained.

To explore the electrochemical reversibility of AB and its derivatives, CV analyses in different supporting electrolytes are conducted (Fig. 1e, f and Supplementary Figs. 2–6). It is found supporting electrolytes including solvents and supporting salts can play an important role in regulating the redox chemistry of AB. AB shows a highly reversible behavior in DMF and ACN electrolytes when using TMABF_4_, TEABF_4_, or tetrabutylammonium hexafluorophosphate (TBAPF_6_) as supporting salts, while it exhibits a quite different electrochemical reversibility when using LiTFSI as supporting salts. In 0.5 M LiTFSI DMF electrolytes, the reversible CV curves of AB are obtained (Fig. 1e), showing a major cathodic peak with a shoulder peak at −1.85 and −1.72 V versus Ag/AgNO_3_, respectively. In contrast, the irreversible electrochemical behavior is observed in A6, LiTFSI DME, and LiTFSI-ACN electrolytes (Supplementary Fig. 2). The redox activity of azo group might be affected by free Li^+^ in some specific solvents such as ACN. In addition, DOL-based supporting electrolytes are also able to support a relatively reversible reaction but with a large potential polarization due to low conductivity or slow reaction kinetics. Moreover, we also investigate the effect of mixed salts (LiTFSI + TEABF_4_) on electrochemical behavior of AB (Supplementary Figs. 3–5). More importantly, at a wide potential range of −3 to 0.5 V, two main cathodic peaks with only one anodic peak are observed in all different electrolytes, indicating the reduction occurred at around −2.5 V versus Ag/AgNO_3_ may be irreversible (Supplementary Fig. 6). This behavior should be referred to a following chemical reaction after the electron transfer^31,32^, which is similar to the protonation process of AB^33^ (Fig. 1d). And due to possible solvation structures existed in LiTFSI DMF electrolytes, lithium ions are probably involved in redox reactions of azo group (Supplementary Fig. 7). Furthermore, we also study the electrochemical properties of HAB and MAB in DMF-based electrolytes (Fig. 1f and Supplementary Figs. 8 and 9). As expected, the protonation effect originated from hydroxy groups severely damages the electrochemical reversibility of HAB while MAB still exhibits a reversible electrochemical behavior similar to AB.

**Fig. 2:**
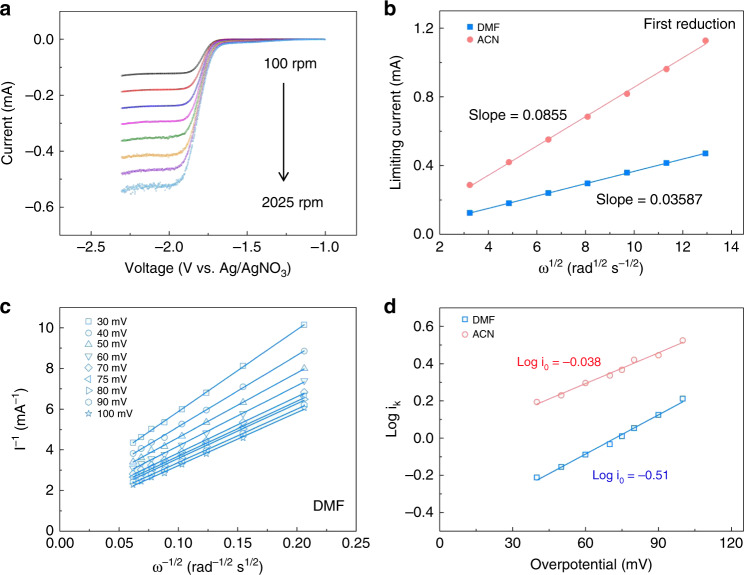
Electrochemical property of AB electrolytes. **a** LSV scans of 10 mM AB in DMF solvents with 0.5 M TEABF_4_. **b** Linearly fitted Levich plots of AB for the first reduction in DMF and ACN supporting electrolytes, respectively. **c** Koutecky–Levich plots of AB in 0.5 M TEABF_4_ DMF supporting electrolytes. **d** Linearly fitted plots based on Butler–Volmer equation as a function of overpotentials.

Further, electrochemical kinetics of AB in DMF or ACN electrolytes were systematically investigated by a three-electrode system with a glassy carbon rotating disk electrode (RDE) (Fig. 2a and Supplementary Fig. 10). In the linear sweep voltammetry, two platforms corresponding to two cathodic reductions are observed, consistent with the CV analysis. As presented in Fig. 2b, the derived slopes can be applied to calculate the diffusion coefficients for these two reductions (*D*_1_/*D*_2_) based on the Levich equation. In the context of one-electron transfer for both the first and second reduction, the calculated diffusion coefficients for DMF and ACN electrolytes are 7.75 × 10^−6^ cm^2^ s^−1^/1.87 × 10^−5^ cm^2^ s^−1^ and 2.85 × 10^−5^ cm^2^ s^−1^/7.23 × 10^−5^ cm^2^ s^−1^, respectively. The diffusion coefficient for the second reduction is slightly higher than the first reduction that might be indicative of reduced molecular size for products of second reduction. And the *D* values of ACN electrolytes are much higher, indicating less solvation effect, which accords with conductivity results. We derived the Koutecky–Levich plots (Fig. 2c) based on the first reduction excluding the second reduction due to its complicated behavior. Using the linear Tafel plot as a function of overpotentials (Fig. 2d), the kinetic rate constant (k_0_) are calculated to be 4.53 × 10^−3^ cm s^−1^ for DMF and 1.34 × 10^−2^ cm s^−1^ for ACN, respectively. As summarized in Supplementary Table 1, the determined diffusion coefficients and rate constants of AB are higher than most inorganic active materials in aqueous systems and comparable to other organic active materials applied in nonaqueous systems.

### Electrochemical performance for NARFBs

In order to further explore the influence of supporting electrolytes on the electrochemical performance of AB, we perform the galvanostatic charging and discharging of 0.1 M AB in various supporting electrolytes (Supplementary Fig. 11). In accordance with the CV results, due to the possible parasitic reactions, it is difficult to achieve the reversible charging and discharging in A6 and LiTFSI-ACN electrolytes. And in ether-based electrolytes (DME, DEGDME, or TEGDME), although the charging and discharging process could be obtained, the serious capacity decay occurs. Moreover, with DOL as solvents, it is able to achieve a relatively stable cycling and the cycling stability of ether electrolytes is also improved with mixed solvents of DOL and DME. In addition, a voltage evolution during initial cycles and considerable voltage polarization are observed, which may be due to instability of LATP at low potentials, limited conductivity, and/or slow reaction kinetics. To eliminate the possible influence caused by Li_1+x+3z_Al_x_(Ti,Ge)_2-x_Si_3z_P_3-z_O_12_ (LATP) and Li_1.5_Al_0.5_Ge_1.5_P_3_O_12_ (LAGP) separators that are stable at low potentials are also tested in DOL electrolytes (Supplementary Fig. 12). However, the similar poor cycling, voltage evolution, and large voltage gap still remain, indicating that the separator is not the key reason dominating the electrochemical performance decay.

**Fig. 3:**
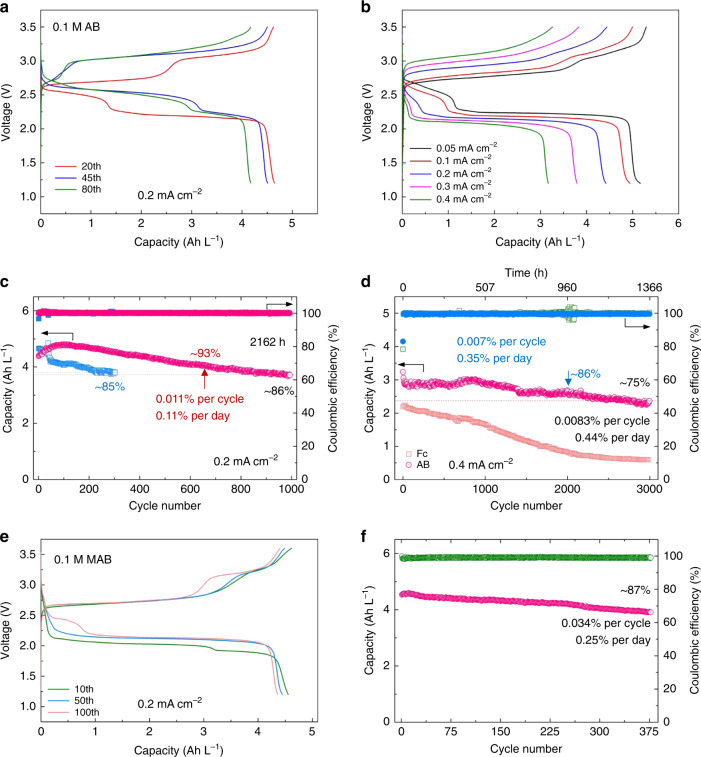
Electrochemical performance of AB and its derivatives at low concentrations. Representative charge and discharge profiles of 0.1 M AB electrolyte at a current density of 0.2 mA cm^−2^ (**a**) and rate performance (**b**). Cycling capacity and coulombic efficiency of 0.1 M AB and 0.1 M Fc electrolytes at a current density of 0.2 (**c**) and 0.4 mA cm^−2^ (**d**), respectively. Charge and discharge profiles (**e**) and cycling performance (**f**) of 0.1 M MAB electrolyte at a current density of 0.2 mA cm^−2^. The supporting salts used in **c** are LiTFSI (blue) and mixed salts (pink, LiTFSI + TEABF_4_).

**Fig. 4:**
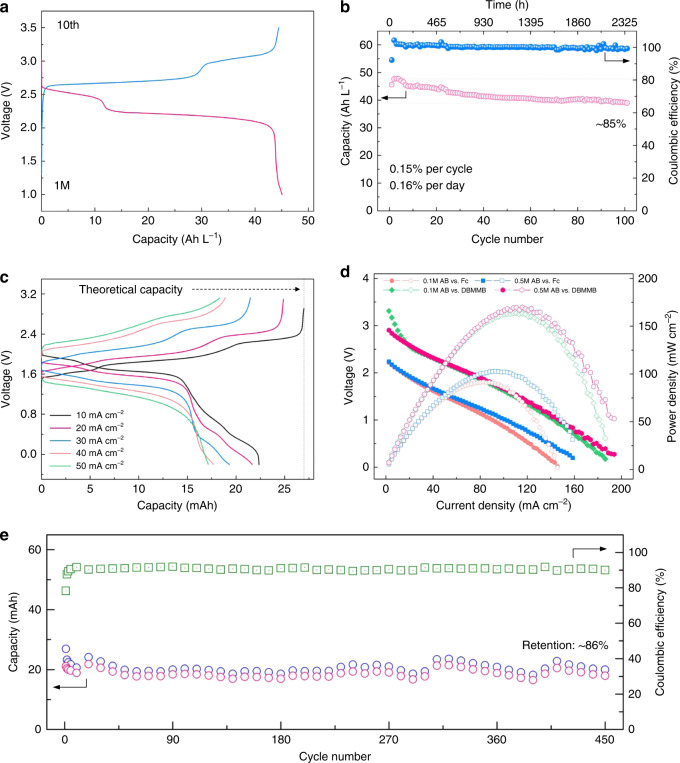
Cycling stability at high concentrations and flow battery test. Representative voltage profile of AB electrolyte (1 M) at the specific cycle at the current density of 0.2 mA cm^−2^ (**a**) and corresponding cycling capacity and coulombic efficiency of 1 M AB electrolyte (**b**). **c** Charge and discharge profiles of 0.1 M AB/Fc flow cell at current densities varied from 10 to 50 mA cm^−2^. **d** Polarization curves and power density of 0.1 and 0.5 M AB/Fc or AB/DBMMB flow cells. **e** Cycling capacity and coulombic efficiency of the 0.1 M AB/Fc flow cell using mixed electrolytes cycled at 25 mA cm^−2^.

**Fig. 5:**
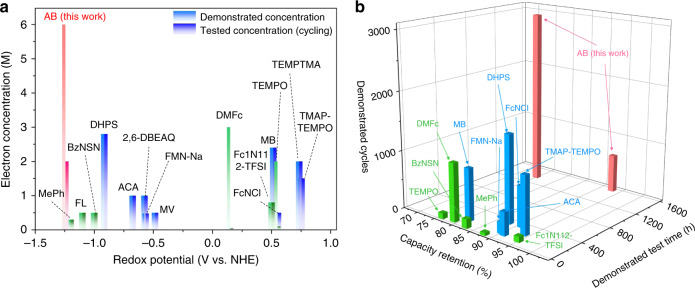
Performance comparison. **a** Comparison of equivalent electron concentration demonstrated in electrochemical tests for AB (red) and various organic molecules in aqueous (blue) or nonaqueous (green) RFBs. **b** Comparison of demonstrated cycles and corresponding test time and capacity retention of AB (red) and various organic molecules in aqueous (blue) or nonaqueous (green) RFBs. DHPS (7,8-dihydroxyphenazine-2-sulfonic acid), 2,6-DBEAQ (4,4′-((9,10-anthraquinone-2,6-diyl)dioxy)dibutyrate), ACA (alloxazine 7/8-carboxylic acid), FMN-Na (flavin mononucleotide), MV (methyl viologen), FcNCl ((ferrocenylmethyl)trimethylammonium chloride), MB (methylene blue), TEMPTMA (N,N,N-2,2,6,6-heptamethylpiperidinyl oxy-4-ammonium chloride), TMAP-TEMPO (4-[3-(trimethylammonio)propoxy]-2,2,6,6-tetramethylpiperidine−1-oxyl chloride), MePh (N-methylphthalimide), FL (9-fluorenone), BzNSN (2,1,3-benzothiadiazole), DMFc (1,1-dimethylferrocene), TEMPO (2,2,6,6-tetra-methylpiperidine-1-oxyl), Fc1N112-TFSI (ferrocenylmethyl dimethyl ethyl ammonium bis(trifluoromethanesulfonyl)imide).

Inferred from the CV measurement, DMF as the chosen solvent is also capable to afford a long stable cycling at both low and high concentrations (Figs. 3 and 4). For practical RFBs, both durability and energy density are important performance parameters when assessing new organic molecules as promising active materials. Specifically, in certain nonaqueous organic RFBs, performance degradation is usually caused by crossover of redox species, radical side reactions, and/or poor chemical stability in organic solvents. Solid ceramic separators are used to assess the potential of AB by eliminating the crossover. And the chemical stability of AB over long periods in supporting electrolytes is confirmed by identical CV curves before and after standing in glove box for over 30 days (Supplementary Fig. 13). More importantly, excellent electrochemical stability is also demonstrated by repeated charging and discharging of 0.1 M AB electrolytes at a current density of 0.2 mA cm^−2^ (Fig. 3a and Supplementary Fig. 14). The voltage profiles at various current densities are presented in Fig. 3b, demonstrating a high utilization of over 95% at 0.05 mA cm^−2^. Moreover, the obvious evolution of voltage profiles during the cycling is found, finally enabling a voltage increase of nearly 0.8 V, which might be related to lithium-coupled redox chemistry. This phenomenon was not observed in the study of alkali-ion batteries^34,35^. But impressively, long-term cycling of 0.1 M AB electrolytes with a coulombic efficiency of over 99.5% is still achieved for 1000 cycles at 0.2 mA cm^−2^, retaining 86% of initial discharge capacity (Fig. 3c). In the first 650 cycles, the battery exhibits a remarkably low capacity fade of 0.011% per cycle and 0.11% per day. What is more, at a higher current density of 0.4 mA cm^−2^, extended cycling stability is further realized with only 25% capacity loss over 3000 cycles, giving a loss rate of 0.0083% per cycle and 0.44% per day (Fig. 3d). Based on the same condition, it is even much better than ferrocene (Fc) that is considered as a stable redox-active molecule in nonaqueous systems, and Supplementary Fig. 15 compares some of the important molecular parameters for AB and competing traditional redox-active molecules including Fc and quinones, suggesting AB is promising for potential NARFBs application. As summarized in Fig. 5 and Supplementary Tables 2 and 3, the unprecedented cycling stability of AB-based NARFBs exceeds previously demonstrated NARFBs and is comparable with some current state-of-the-art ARFBs in terms of time-based fade rate. Furthermore, realizing a long cycling life at high concentrations (≥1 M) is becoming more and more important for practical applications. In Fig. 4, we demonstrate that the assembled battery using 1 M AB electrolytes (equivalent to 2 M electron transfer) delivers a reversible capacity of ~46 Ah L^−1^ (~86% utilization) with only 15% capacity loss for cycling over three months (fade rate 0.15% per cycle, 0.16% per day over 100 cycles) at 0.2 mA cm^−2^. It is also worth mentioning that the battery is successfully operated even at a higher concentration of 2 and 3 M, respectively, producing a high capacity of up to 138 Ah L^−1^ (Supplementary Fig. 16). But the precipitation in 2 or 3 M electrolytes observed in the disassembled battery results in a poor cyclability (Supplementary Fig. 17), which coincides with the precipitation of 2 M AB in 2 M LiTFSI DMF solutions. Furthermore, in order to show the potential for practical application, we also conducted the flow battery test by pairing AB with Fc or DBMMB (Fig. 4 and Supplementary Figs. 18–21). With a porous membrane and mixed electrolytes, the demonstrated AB/Fc flow battery exhibits reasonable capacity utilization and efficiency results under various current densities (10–50 mA cm^−2^) and achieves a peak power density of 103 mW cm^−2^. When using DBMMB as the positive species, the maximum power density can reach 170 mW cm^−2^, which is higher than most of reported NARFBs and comparable to some demonstrated aqueous organic RFBs (Supplementary Fig. 22). At a current density of 25 mA cm^−2^, the AB/Fc flow battery shows a stable cycling at both low (0.1 M) and high (0.4 M) concentrations. With 0.1 M AB and Fc mixed electrolytes, the capacity retains ~86% of the initial value after 450 cycles. And based on our previous study, the optimization of electrolyte design such as a biredox strategy may have the potential to further improve the cycling demonstration in NARFBs^16^.

We further explore the effect of functional groups on the cycling stability of AB. Identical to CV tests, the hydroxy group adversely affects the electrochemical stability of AB possibly due to the protonation effect while MAB with methoxy groups still maintains the excellent cycling stability with a similar evolution of voltage profiles under cycling (Fig. 3e, f and Supplementary Fig. 14). The cut-off voltage also plays a role in cycling stability (Supplementary Fig. 23) because of possible side reactions at high potentials. As discussed above, Ti^4+^ of LATP separators could be reduced at low potentials^36^, leading to increased resistance as confirmed by the color change of separators and increased voltage polarization at the current density of 0.2 mA cm^−2^ (Supplementary Fig. 24). Nevertheless, when the current density is reduced to 0.05 mA cm^−2^, the voltage profiles are gradually resumed to the original state because of the excellent cycling stability, which reflects the nature of AB electrolytes. And when using LAGP separators that are stable at low potentials, similar electrochemical performance with a prolonged cycling life is achieved (Supplementary Fig. 25), further confirming the potential of AB for high-performance NARFBs.

### Lithium-coupled reaction mechanism

**Fig. 6:**
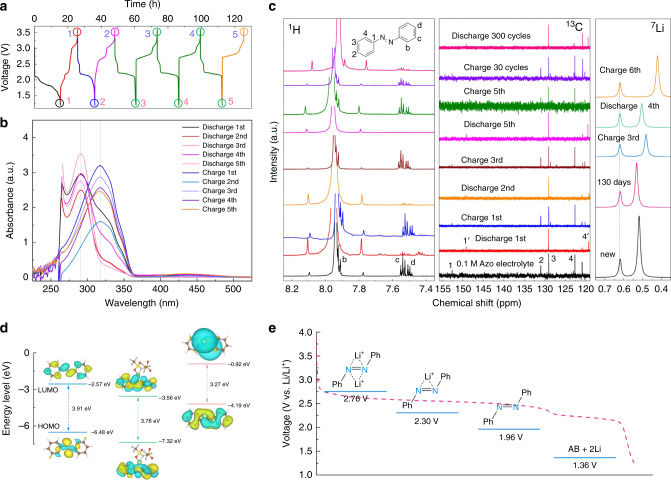
Characterization of electrochemical reversibility and reaction mechanism. Charge and discharge profiles of 0.1 M AB electrolyte over time at the current density of 0.05 mA cm^−2^ (**a**) and corresponding UV–vis spectra recorded at different charge and discharge state (**b**). **c** ^1^H, ^13^C, and ^7^Li NMR spectra of 0.1 M AB electrolyte measured at various selected charge and discharge cycles. **d** Optimized structure and calculated energy levels of AB, AB-LiTFSI, and Li_2_AB obtained from DFT simulations. **e** Comparison between the discharge voltage curve after the voltage evolution and predicted voltages of AB in different coordination configurations.

To reveal the lithium-coupled redox chemistry and confirm the chemical stability of reduced AB products, ex situ UV–vis and NMR spectra in charging and discharging were conducted together with detailed density functional theory (DFT) simulation (Fig. 6). The charging and discharging profile of 0.1 M AB electrolyte and corresponding ex situ UV–vis spectra at different states are displayed in Fig. 6a, b. The identical spectra corresponding to charging and discharging state, respectively, switch back and forth in the cycling, which confirms the reversible redox activity of AB in LiTFSI DMF electrolytes. After the discharge, the characteristic peak of phenyl rings (π–π* absorption) experiences an obvious blue shift due to the change of electron density stemming from the opening of pi bond in the azo group (N=N), which is in agreement with the statement that azo group is considered as the redox-active center for AB. Consistently, the peak at around 425 nm corresponding to the n–π* absorption of azo group disappears after discharging and recovers after charging (Supplementary Fig. 26), further confirming the excellent electrochemical reversibility of AB. As presented in Fig. 6a, the obvious evolution of voltage profiles is occurred in a few cycles at a low current density of 0.05 mA cm^−2^ when using LiTFSI as supporting salts. The similar phenomenon of positive shifts of average redox potentials was also found in pteridine derivatives^37,38^ and other active materials^39–41^, suggesting a redox activation process of azo group accompanied with Li^+^ coupling because of possible interactions of Li^+^ and nitrogen atoms^42^. Certainly, lithium ions can also interact with oxygen in carbonyl groups of DMF or TFSI anions. Considering the activation process is just triggered after the first discharging, it is reasonable to find the UV–vis spectrum at the first discharging state has a slight difference. In order to further identify the lithium-coupled redox reaction, UV–vis spectra of hydrazobenzene (AB-2H) obtained by the protonation of AB is also tested (Supplementary Fig. 26), which is identical with that of reduced AB, thus confirming the existence of interacting Li^+^. Moreover, the chemical stability of reduced AB and structure stability of AB during the cycling are further investigated by the spectra (Supplementary Figs. 27–29). There is no obvious peak shift in the UV–vis spectra of reduced AB after resting for 5 days in glove box, confirming the good chemical stability of reduced AB in DMF electrolytes. The structural and electrochemical stability of AB are also confirmed by the identical UV–vis spectra at charged state before and after cycling for a long time, respectively. We also study the UV–vis spectra of AB derivatives after cycling test at the charging state (Supplementary Fig. 30), and it is found that HAB shows a totally different curve due to the possible proton-induced structure change while MAB still obtains the similar UV–vis spectra after cycling as the indication of stable molecular structure.

Ex situ NMR analysis also confirms the reversible change in the molecular structure of AB during the lithium-coupled redox reaction (Fig. 6c and Supplementary Figs. 31–33). Taking the NMR solvent (CD_2_Cl_2_) as the inner reference, the standard spectra of AB with or without the supporting salt (bis(trifluoromethane)sulfonimide lithium salt (LiTFSI)), were obtained, showing the characteristic molecular structure (Supplementary Fig. 31). By comparing NMR spectra of AB in different conditions, we find the curves of 0.1 and 1 M AB electrolytes storing for over 30 days are almost identical to that of freshly prepared 0.1 M AB DMF solutions, further confirming the excellent chemical stability of AB. In addition, in the ^1^H-NMR spectra (Supplementary Fig. 32), the chemical shift of AB with DMF solvents exhibits a slight high-field shifting (to lower ppm) for each proton position, indicating the solvation effect of DMF. When LiTFSI as supporting salt is added into the solution, the chemical shift of AB and hydrogen connected to carbonyl groups in DMF shows a slight low-field and high-field shifting, respectively, which causes the overlap of corresponding peaks (Hb and He), implying the possible intermolecular interactions existed in both dilute and concentrated AB electrolytes. On the other hand, the NMR spectra of 0.1 M AB electrolytes during the cycling, as shown in Fig. 6c, demonstrate the reversible molecular change and good structure stability of AB during the redox reaction, which is consistent with UV–vis analysis. A few additional peaks are found in the ^1^H NMR spectra after the first discharging and charging, hinting the chemical environment change, which might be related to the activation process. However, the reversible change of both ^1^H and ^13^C NMR spectra after discharging and charging clearly proves the excellent electrochemical stability and reversibility of AB. And it is further noted that the change of π-conjugated structure after discharging, due to the reduction of azo group, can be reflected in the ^13^C NMR spectra, leading to the significant peaks shifting of C1 and C4 towards high field (C1′ and C4′). Considering the capacity decay after the long cycling, some tiny peaks may appear because of slight side reaction of AB molecules. But the excellent electrochemical stability is still confirmed by the nearly identical NMR spectra after a long cycling compared to the original electrolyte. Although after the long cycling Besides, the small chemical shift (<1 ppm) observed in both ^1^H and ^13^C NMR spectra of DMF during the discharging and charging (Supplementary Figs. 33 and 34) could be also a hint of lithium-coupled redox chemistry with a solvation structure in DMF. We also characterize the NMR spectra of AB in LiTFSI DOL electrolytes before and after electrochemical test (Supplementary Fig. 35), but no such special intermolecular interaction is detected as indicated by the negligible change in the NMR spectra.

The lithium-coupled redox chemistry of AB accompanied with voltage evolution is further supported by ^7^Li NMR spectra and DFT simulations. The energy levels of the highest occupied molecular orbital (HOMO) and lowest unoccupied molecular orbital of AB, AB-LiTFSI, and reduced Li_2_AB are first calculated as displayed in Fig. 6d. When LiTFSI is coordinated with azo group, the HOMO plot shows a more effective electron delocalization in the conjugated structure, suggesting the interaction between LiTFSI and azo group can further improve the structural stability, which is consistent with the result of nucleus independent chemical shift (NICS). The average NICS of the AB-LiTFSI configuration (−7.3 ppm) is lower than AB (−6.5 ppm), indicating an enhanced aromaticity. Note that the aromaticity of reduced product, Li_2_AB (NICS = −4.2 ppm), is still kept, suggesting good structural stability during redox reactions. In addition, the gap of HOMO energy levels between AB and Li_2_AB increases when LiTFSI interacts with azo group, leading to a larger energy barrier for redox reactions, indicating a possible voltage increase. Thus, we propose that the interaction between lithium cations and azo group should be the reason for the voltage evolution during the cycling. In order to gain insight into reaction mechanism, ^7^Li NMR is measured to reveal the possible coordination between Li^+^ and azo group (Fig. 6c). With the help of an external reference (1 M LiTFSI DMF, dash line), the chemical shifts of 0.1 M AB electrolytes before and after standing for about 4 months confirm the change of chemical environment of Li^+^ after adding the AB molecule. Since azo group represents a less electronegative ligand, compared to carbonyl or sulfoxide groups in DMF or TFSI^−^, respectively, it is consistent to find a more negative chemical shift (ppm) when Li^+^ interacts with azo group, leading to a shielded environment of Li nucleus^43^. Thus, it verifies the existing coordination of Li^+^ and azo group in the original electrolyte. However, along with charging and discharging, the continuous change of ^7^Li chemical shift suggests the dynamic evolution of Li^+^ coordination environment, which is in accordance with the continuous evolution of voltage profiles. To further confirm the lithium-coupled redox chemistry, we compare simulated voltages of possible AB configurations with the discharge curve after the voltage evolution (Fig. 6e). It is found that the simulated voltages of AB-LiTFSI and AB-2LiTFSI configurations coincidently matches well with two major voltage plateaus in the discharge curve, confirming the lithium-coupled redox reactions. As the voltage profiles continuously evolves during the cycling (Supplementary Fig. 14a), we infer that azo group is partially coordinated by one Li^+^ in original electrolytes and it dynamically changes to the final coordination with two Li^+^ during the cycling. These results agree with the ex situ UV–vis and NMR analysis and confirm that the azo group works as a collaborative center for the lithium-coupled redox chemistry.

## Discussion

Based on the redox activity of azo group, the AB molecule is demonstrated as a promising redox-active organic material for high-performance NARFBs with high energy density and long cycle life. As the base aromatic azo compound, AB is commercially available in large scale and plenty of derivatives are known as abundant dyes in industry. With the *trans*-structure, AB is stable at ambient environment and shows a high solubility in common organic solvents. When using DMF-based supporting electrolytes, it shows a long stable cycling with a capacity retention of 99.992% per cycle over 3000 cycles and functions well at high concentrations with energy density of ~101 Wh L^−1^. AB also exhibits the lithium-coupled redox chemistry accompanied with a voltage increase of ~0.8 V, due to the interaction of Li^+^ and azo group as confirmed by ^7^Li NMR characterizations and DFT calculations. Nevertheless, more study is still needed for the detailed underlying mechanism, such as the solvation configuration and detailed Li^+^ coordination steps.

We also explored the potential of molecular functionalization on azo compounds for tailoring the solubility, redox potential, and stability, demonstrating that methoxy group can improve the solubility in ACN and maintain the electrochemical activity of AB while functional groups as proton donors may damage the electrochemical reversibility of azo group. This work presents a new aspect in designing promising organic electrolytes with high stability and capacity, and shows great potential of azo-aromatic organics as redox-active species to accelerate the development of next-generation RFBs. However, when compared to the commercialized vanadium-based RFBs, more research efforts are still needed to further improve the stability of organic molecules in nonaqueous electrolytes in order to make it possible for practical applications.

## Methods

### Chemicals and materials

AB, HAB, AB-2H, polyvinylidene fluoride (PVDF) binder, and N-methyl-2-pyrolidone (NMP) were obtained from Fisher Scientific. MAB, TMABF_4_, Fc, TEABF_4_, TBAPF_6_, LiTFSI, and all used organic solvents except for EC were from Sigma-Aldrich. Super P carbon black was received from Timcal Graphite & Carbon. EC and Li-ion electrolyte (A6, 1 M LiPF_6_ in EC/diethyl carbonate (DEC) with volume ratio of 1:1) were purchased from BASF. DBMMB was obtained from Pacific Northwest National Laboratory. All chemicals were used as received without any further treatment or purification. The NASICON-type LATP ceramic separator was received from Ohara Corporation. The LAGP ceramic separator was received from MTI Corporation. Daramic^®^ porous separators were obtained from Daramic LLC (Owensboro KY). The dissolution of active materials was conducted in glove box at room temperature.

### Characterizations

The UV–vis spectra measurement was performed on a UV–vis spectrometer (Evolution 300, Thermo Scientific) from 200 to 800 nm. For the ex situ measurement of catholytes at various charging and discharging state, the tested battery was first transferred into glove box and all the samples were carefully sealed by the tape to avoid the influence of air and moisture. ^1^H and ^13^C NMR measurements were carried out with a 600 MHz Varian VNMRS instrument. The ^7^Li NMR spectra were measured on a 400 MHz Varian instrument in 5 mm NMR tubes with an external reference in the form of a sealed, coaxial capillary containing a certain amount of 1 M LiTFSI DMF solutions in CD_2_Cl_2_. In NMR test, all the samples were dissolved in dichloromethane-d_2_. Similar to the UV–vis measurement, the sample preparation was conducted under the protection of Ar gas and the careful sealing was taken during the transfer and test.

### Electrochemical measurement

The ionic conductivities of different supporting electrolytes were measured by the electrochemical impedance spectra using two glassy carbon electrodes. The CV measurements were conducted on the Autolab (PGSTAT302N) electrochemical workstation and RDE measurements were conducted using a BioLogic RRDE-3A rotating ring disk electrode. Both CV and RDE test used the three-electrode configuration with glassy carbon as working electrode (diameter 3 mm), Ag/AgNO_3_ (0.01 M) in ACN as reference electrode and Pt wire as counter electrode. The scan rates in CV tests were 10, 20, 50, 100, and 200 mV s^−1^. In RDE tests, the working current was recorded in the potential range from −1 to −3 V versus Ag/AgNO_3_ (0.01 M) at 5 mV s^−1^. The rotation rates were 100, 225, 400, 625, 900, 1225, 1600, 2025, 2500 r.p.m. The diffusion coefficient (*D*) was calculated according to Levich equation: *i*_*lim*_ = 0.62*nFAD*^2/3^*ω*^1/2^*ν*^−1/6^*C*, where *i*_*lim*_ is limiting current, *n* is the number of electrons transferred, *F* is the Faraday constant (96485 C mol^−1^), *A* is the surface area of the working electrode (0.0707 cm^2^), *C* is molar concentration in 1 × 10^−5^ mol cm^−3^, *ν* is the kinetic viscosity in cm^2^ s^−1^ (9.7 × 10^−3^ and 1.0 × 10^−2^ cm^2^ s^−1^ for DMF- and ACN-based solutions) and *ω* is the routing angular velocity in rad s^−1^. The Koutecky–Levich plots at different overpotentials were extrapolated to get the kinetic current i_k_ according to the Koutecky–Levich equation:

$$1/i = 1/i_k + 1/\left( {0.62nFAD^{2/3}\omega ^{1/2}\nu ^{ - 1/6}C} \right)$$

The exchange current (*i*_*0*_) can be obtained by fitting *i*_*k*_ to the Tafel plot at the overpotential of zero, from which the reaction rate constant (*k*_*0*_) was determined according to the Butler–Volmer equation: *i*_*0*_ = *nFCk*_*0*_.

### Battery assembly and test

The similar battery assembly procedure can be also found in our previous reports. The first step is to make the cylinder cell, sealing two cylindrical quartz shells (inner/outer diameters are 8/14 mm) by sandwiching the LATP or LAGP separator with Surlyn^®^ resin sealant and heating at 120 °C for 2 h. Then, the as-prepared Super P/PVDF (90:10 wt%) slurry using NMP solvents was coated onto the surface of Ti foil as current collectors for the cathode side, and a tiny hole was drilled for injecting the catholyte. In the anode part, Li metal was pressed onto the copper mesh attached to the copper foil as current collectors. Next, the cathode current collector and previous cylinder cell were bonded together using sealant. Lastly, we added electrolytes to both the anode and cathode sides before testing. Commercial A6 electrolyte was injected into the quartz shell of the anode part, and then sealed hermetically using epoxy. In the cathode, azo compounds dissolved in supporting electrolytes were injected into the cylindrical quartz shell through the hole drilled in the Ti foil then immediately covered by Kapton tape.

All battery tests were performed on a BioLogic VMP3 potentiostat, Autolab, or LAND system at room temperature. At low-concentration battery tests, the applied supporting electrolyte is generally 0.5 M LiTFSI dissolved in DMF solvents. In addition, mixed supporting salts (LiTFSI and TEABF_4_) were also utilized to study the cycling performance of 0.1 M AB electrolytes. At high-concentration battery tests, the applied supporting electrolyte is 1 M LiTFSI in DMF solvents. For electrochemical tests at 2 or 3 M, considering the tranfer of charge carriers (Li^+^) from anode to cathode side, the concentration of LiTFSI in orignal catholytes was reduced to 0.5 and 0.25 M, respectively. In general, the cut-off potential was set between 1.2 and 3.5 V. And in most cases, the LATP ceramic membrane (150 μm) was used as the separator to study the battery performance. Besides, the LAGP ceramic separator was also used for battery tests in a few specific performance demonstrations. The capacity and energy density were calculated based on the single electrolyte, leading to a theoretical capacity of 5.36 Ah L^−1^ at 0.1 M (two electrons transferred). The flow tests were carried out in the cell comprised of poly(tetrafluoroethylene) frame, graphite plates current collector, and graphite felt electrodes with an active area of 4 cm^2^. Daramic 175^®^ was used as the separator and the electrolytes were circulated at a flow rate of 30 ml min^−1^. For AB/Fc flow cell tests, 0.1 M AB +0.15 M Fc or 0.4 M AB +0.2 M Fc mixed electrolytes (5 ml) were used as the anolyte. For AB/DBMMB flow cell tests, 0.1 M AB +0.2 M DBMMB or 0.5 M AB +1 M DBMMB in LiTFSI DME mixed electrolytes were used as the catholyte and 0.1 M AB or 0.5 M AB in LiTFSI DMF electrolytes (5 ml) were used as the corresponding anolyte, respectively.

### DFT simulation

Electron configurations of the molecules were calculated by DFT method within the framework of the Gaussian 09 package. The standard Pople basis set, 6–311G++(d,p), combined with the Lee–Yang–Parr exchange correlation functional (B3LYP) was used for all calculations. Various kinds of geometry for the negatively charged AB, Li_*n*_AB (*n* = 1, 2) and LiTFSI coordinated AB were explored, and the most energy favorable geometry is selected. For each combination, the geometry was fully optimized to achieve the lowest total energy before energy level calculation, and all possible spin multiplicities were explored (*S* = 1–4), among which we chose the one with the lowest energy for analysis and comparison between different combination.

## Supplementary information

Supplementary Information

## Data Availability

The authors declare that all data supporting the findings of this study are included within the paper and its Supplementary Information files. Source data are available from the corresponding author upon reasonable request.
