# Supplementary material for: Reversible redox chemistry in azobenzene-based organic molecules for high-capacity and long-life nonaqueous redox flow batteries
Source: Nat Commun. 2020 Jul 31;11:3843. doi: 10.1038/s41467-020-17662-y (PMC7395718; doi:10.1038/s41467-020-17662-y)
Supplement: Supplementary file 1 — Supplementary Information [file 41467_2020_17662_MOESM1_ESM.pdf]

Supplementary Information

Reversible redox chemistry in azobenzene-based organic molecules for high-capacity and long-life nonaqueous redox flow batteries

Zhang *et al.*

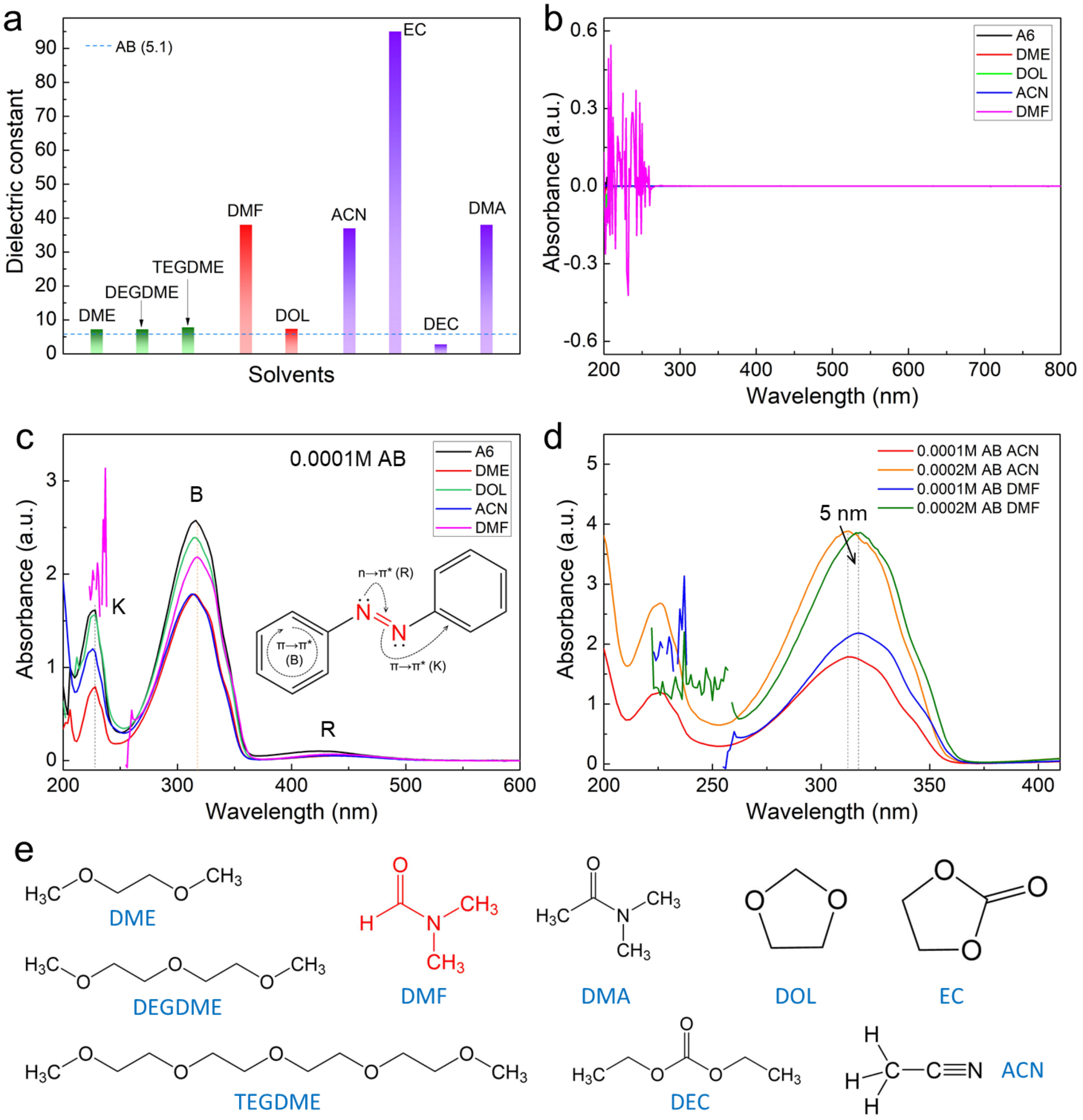

Supplementary Figure 1. a, Dielectric constants of selected solvent molecules. **b**, UV-vis spectra of various organic solvents at room temperature. **c**, UV-vis spectra of AB dissolved in various organic solvents and corresponding peak assignment based on the molecular structure of AB (inset). **d**, UV-vis spectra of AB in DMF and ACN solvents. **e**, Molecular structures of different selected organic solvents.

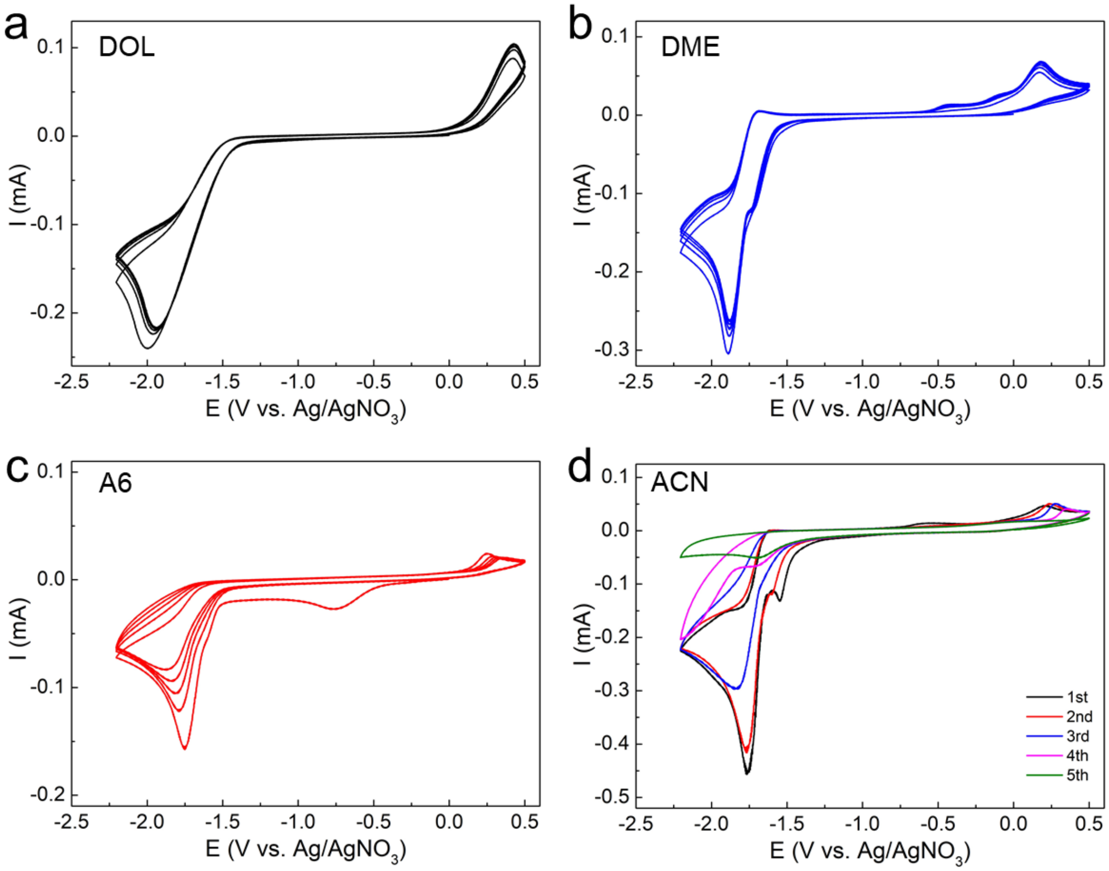

Supplementary Figure 2. a,**b**,**c**,**d**, CV curves of 10 mM AB in DOL (**a**), DME (**b**), A6 (**c**) and ACN (**d**) supporting electrolytes at the scan rate of 100 mV s^{-1}, respectively. In A6 electrolytes, 1M $LiPF_6$ is used as supporting salts and in other three supporting electrolytes, 0.5M LiTFSI is used as supporting salts.

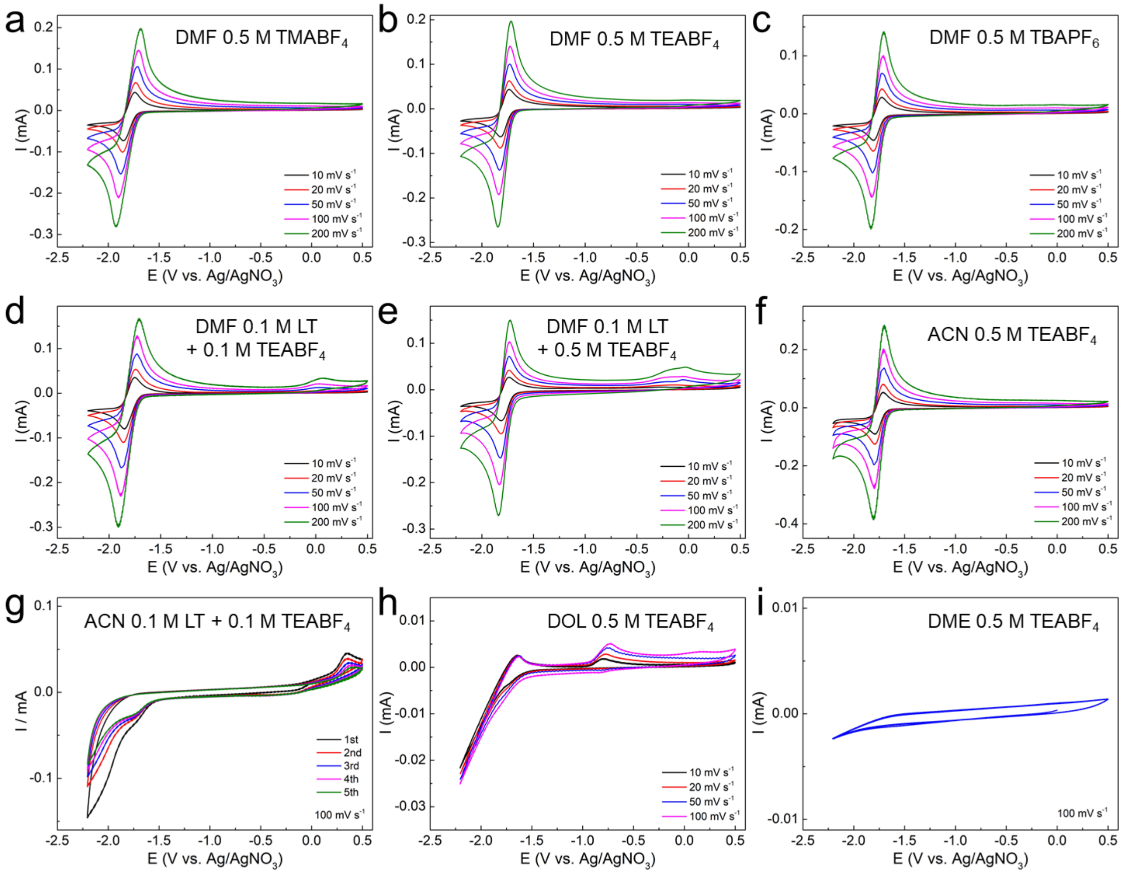

Supplementary Figure 3. a,**b**,**c**,**d**,**e**, CV curves of 10 mM AB in DMF-based supporting electrolytes at various scan rates. **f**,**g**, CV curves of 10 mM AB in ACN-based supporting electrolytes at various scan rates. **h**, CV curves of 10 mM AB in 0.5 M $TEABF_4$ DOL supporting electrolytes at various scan rates. **i**, CV curves of 10 mM AB in 0.5 M $TEABF_4$ DME supporting electrolytes at a scan rate of 100 mV s^{-1}. $TEABF_4$ supporting salts are nearly insoluble in DOL or DME solvents.

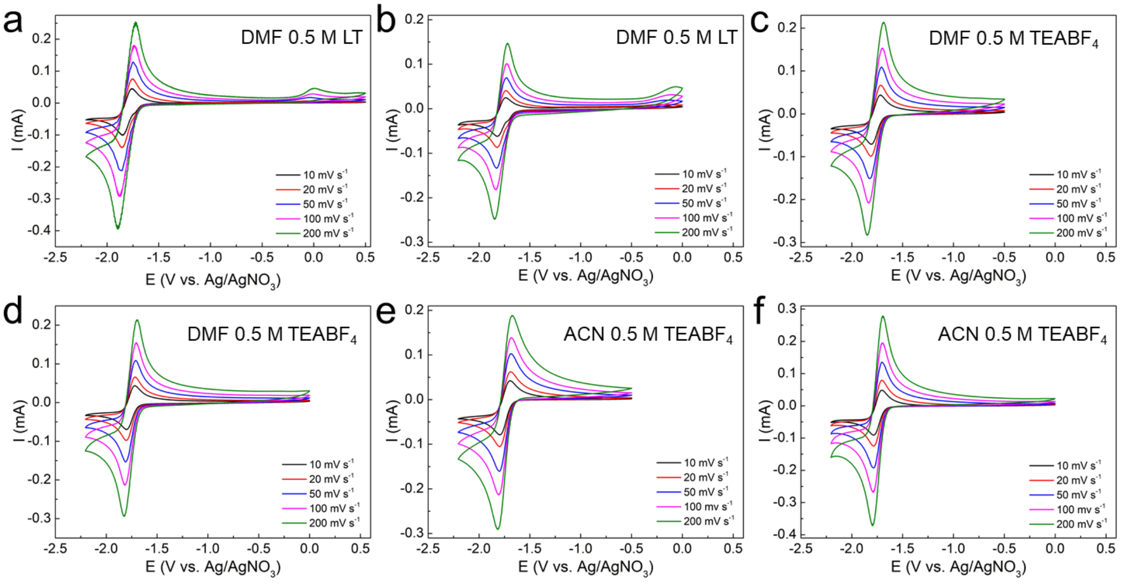

Supplementary Figure 4. a,**b**, CV curves of 10 mM AB in 0.5 M LiTFSI DMF supporting electrolytes at various scan rates. **c**,**d**, CV curves of 10 mM AB in 0.5 M $TEABF_4$ DMF supporting electrolytes at various scan rates. **e**,**f**, CV curves of 10 mM AB in 0.5 M $TEABF_4$ ACN supporting electrolytes at various scan rates.

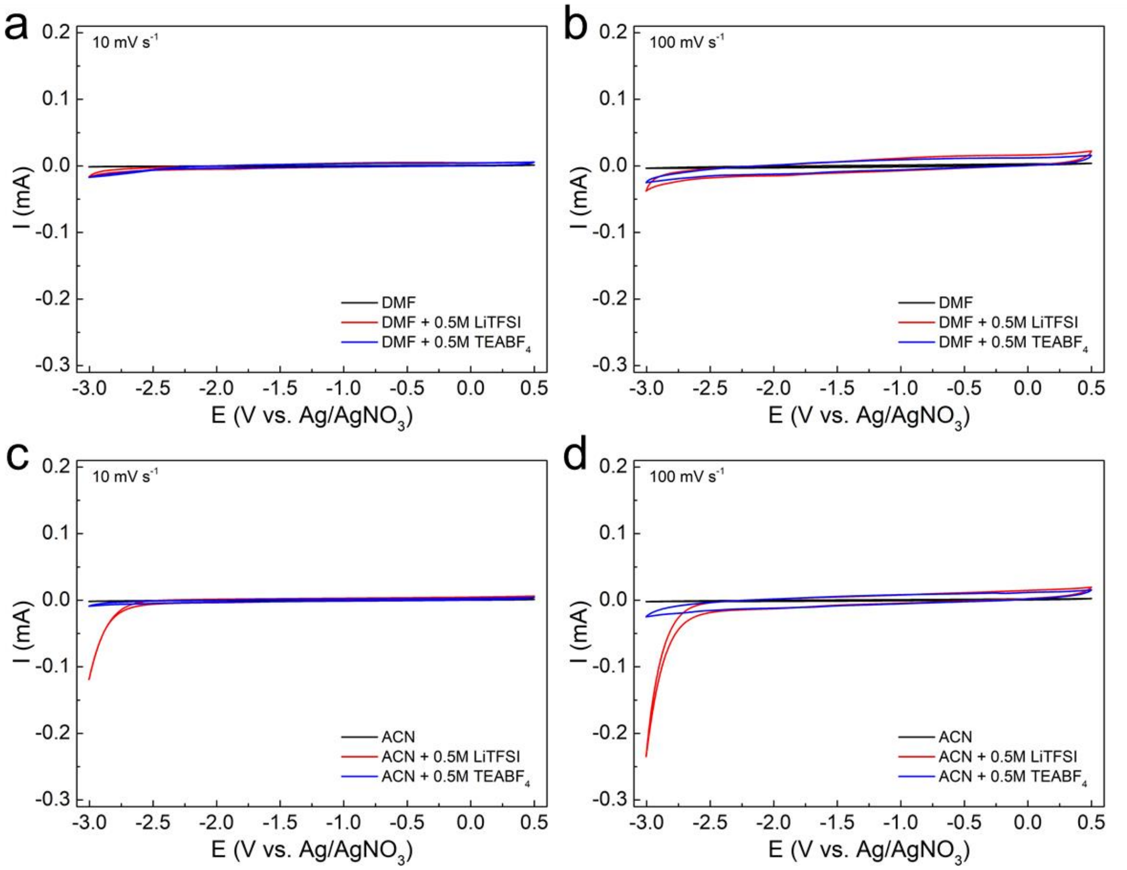

Supplementary Figure 5. **a**,**b**, CV curves of DMF solvents and DMF-based supporting electrolytes at the scan rates of 10 (**a**) and 100 mV s^{-1} (**b**). **c**,**d**, CV curves of ACN solvents and ACN-based supporting electrolytes at the scan rates of 10 (**c**) and 100 mV s^{-1} (**d**).

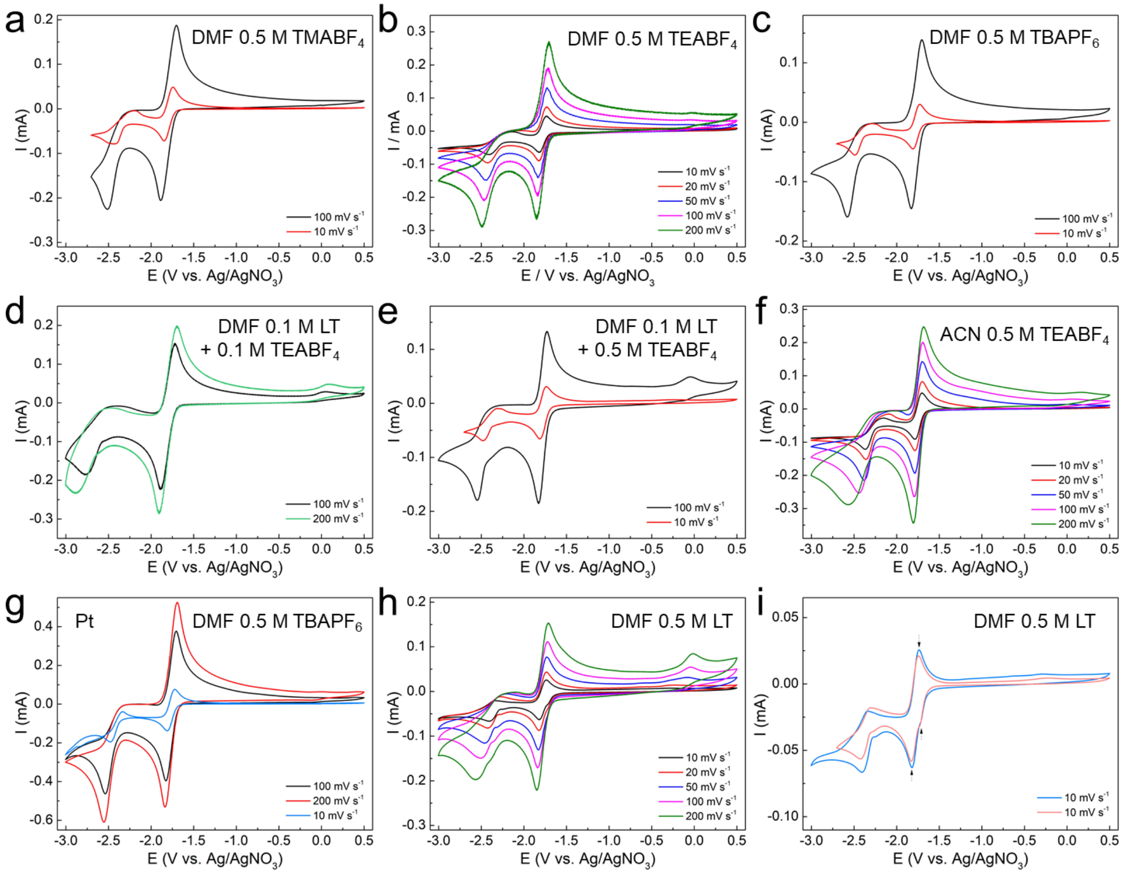

Supplementary Figure 6. **a**,**b**,**c**,**d**,**e**, CV curves of 10 mM AB in DMF-based supporting electrolytes at various scan rates. **f**, CV curves of 10 mM AB in ACN-based supporting electrolytes at various scan rates. **g**, CV curves of 10 mM AB in 0.5 M $TBAPF_6$ DMF supporting electrolytes using Pt working electrode at various scan rates. **h**, CV curves of 10 mM AB in 0.5 M LiTFSI DMF supporting electrolytes at various scan rates. **i**, CV curves of 10 mM AB in 0.5 M LiTFSI DMF supporting electrolytes at a scan rate of 10 mV s^{-1}. In all other CV tests except for (**g**), the applied working electrode is glassy carbon.

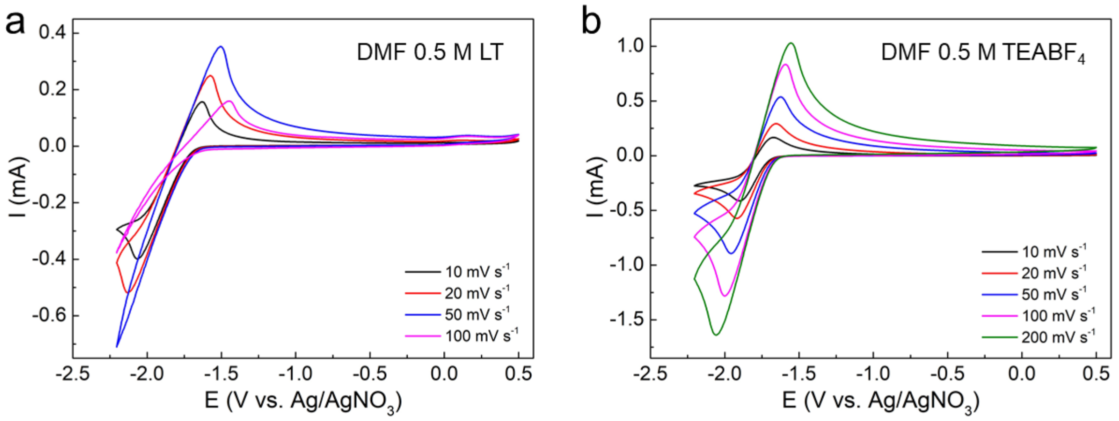

Supplementary Figure 7. a,**b**, CV curves of 100 mM AB in DMF-based supporting electrolytes with 0.5 M LiTFSI (**a**) or $TEABF_4$ (**b**) at various scan rates.

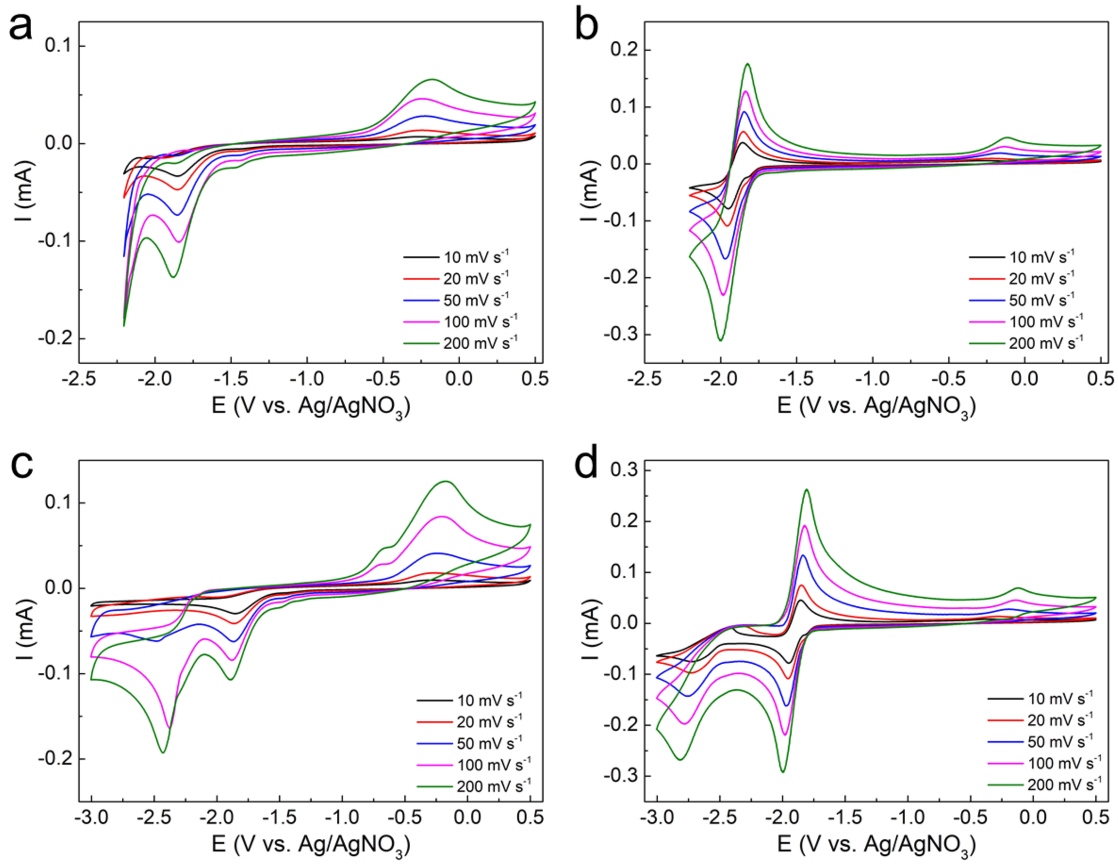

Supplementary Figure 8. a,**b**, CV curves of 10 mM HAB (**a**) and 10 mM MAB (**b**) in 0.5 M LiTFSI DMF electrolytes at various scan rates. **c**,**d**, CV curves of 10 mM HAB (**c**) and 10 mM MAB (**d**) in 0.25 M LiTFSI DMF electrolytes at a wide potential range (-3.0~0.5 V) at various scan rates.

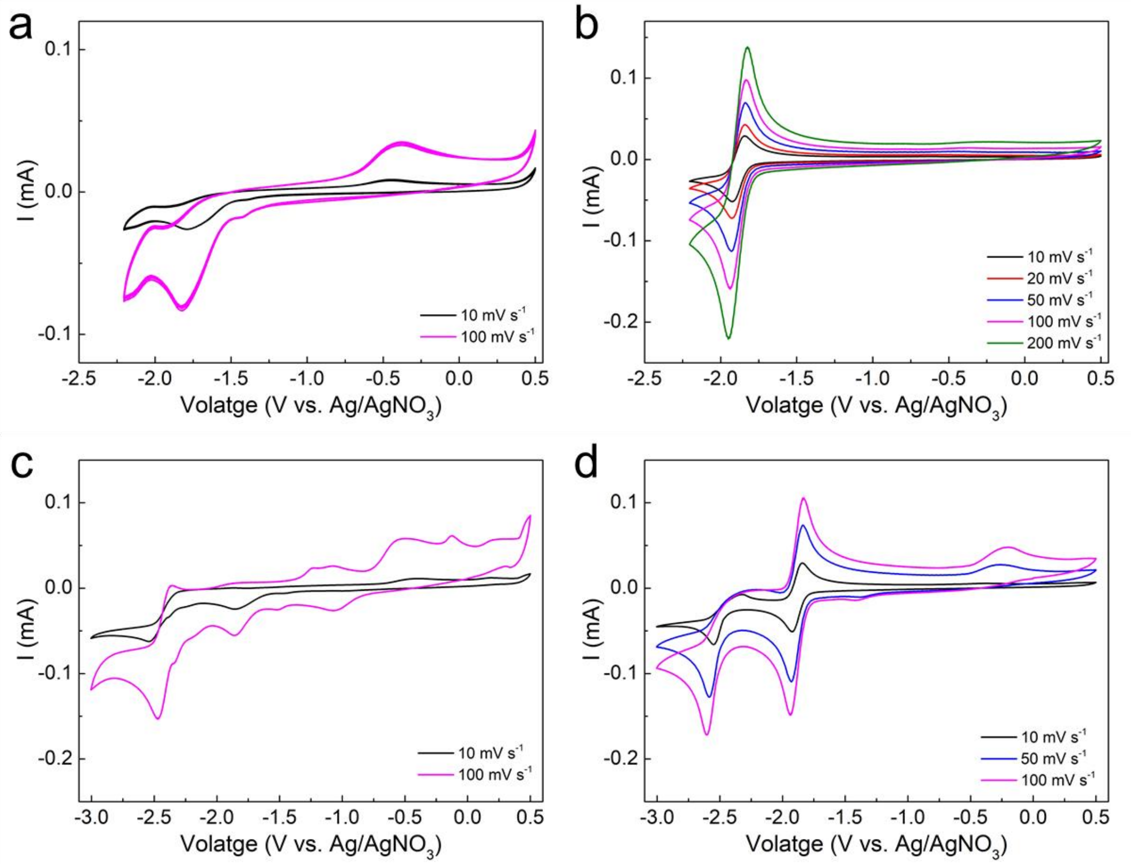

Supplementary Figure 9. **a**,**b**, CV curves of 10 mM HAB (**a**) and 10 mM MAB (**b**) in 0.25 M $TEABF_4$ DMF electrolytes at various scan rates. **c**,**d**, CV curves of 10 mM HAB (**c**) and 10 mM MAB (**d**) in 0.25 M $TEABF_4$ DMF electrolytes at a wide potential range (-3.0~0.5 V) at various scan rates.

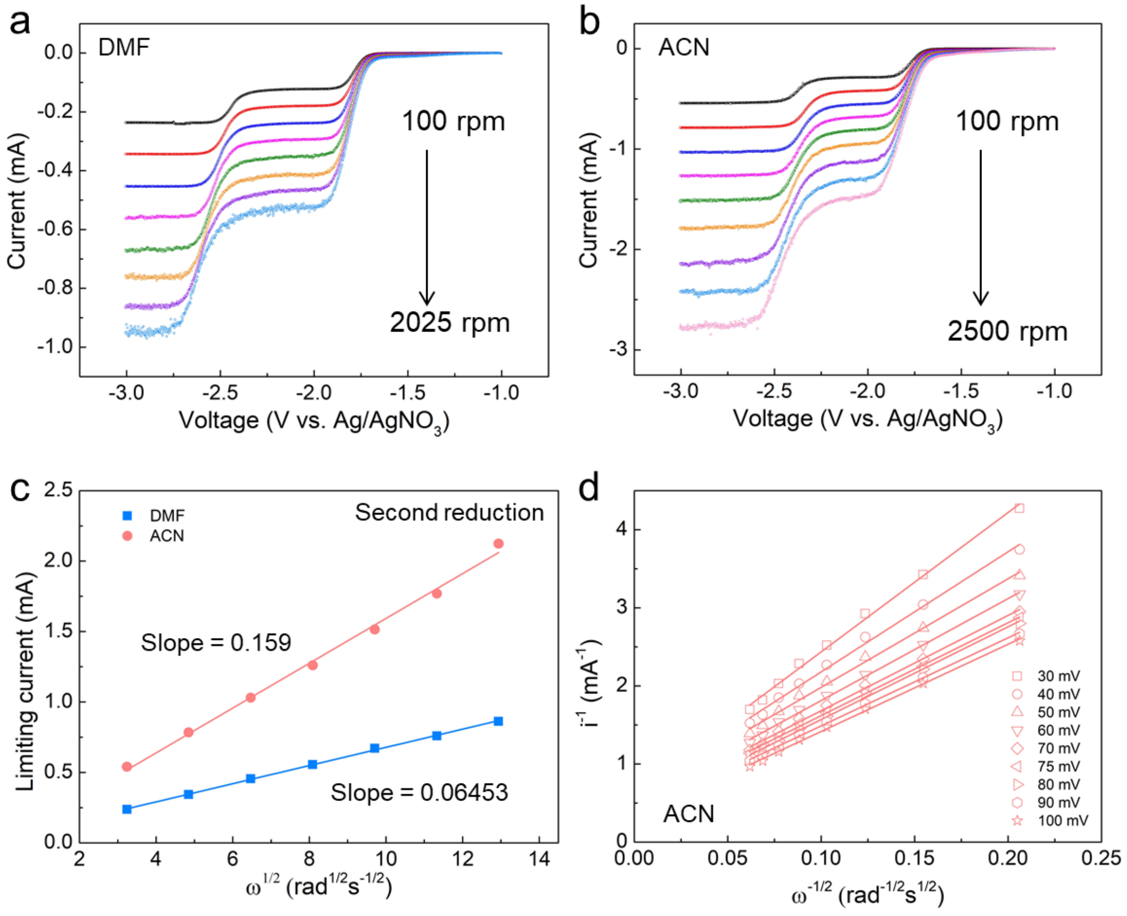

Supplementary Figure 10. a,**b**, LSV scans of 10 mM AB in 0.5 M $TEABF_4$ DMF (**a**) or 0.5 M $TEABF_4$ ACN (**b**) electrolytes with a rotating working electrode. **c**, Linearly fitted Levich plots of AB for the second reduction in DMF and ACN supporting electrolytes, respectively; **d**, Koutecky-Levich plots of AB in 0.5 M $TEABF_4$ ACN supporting electrolytes.

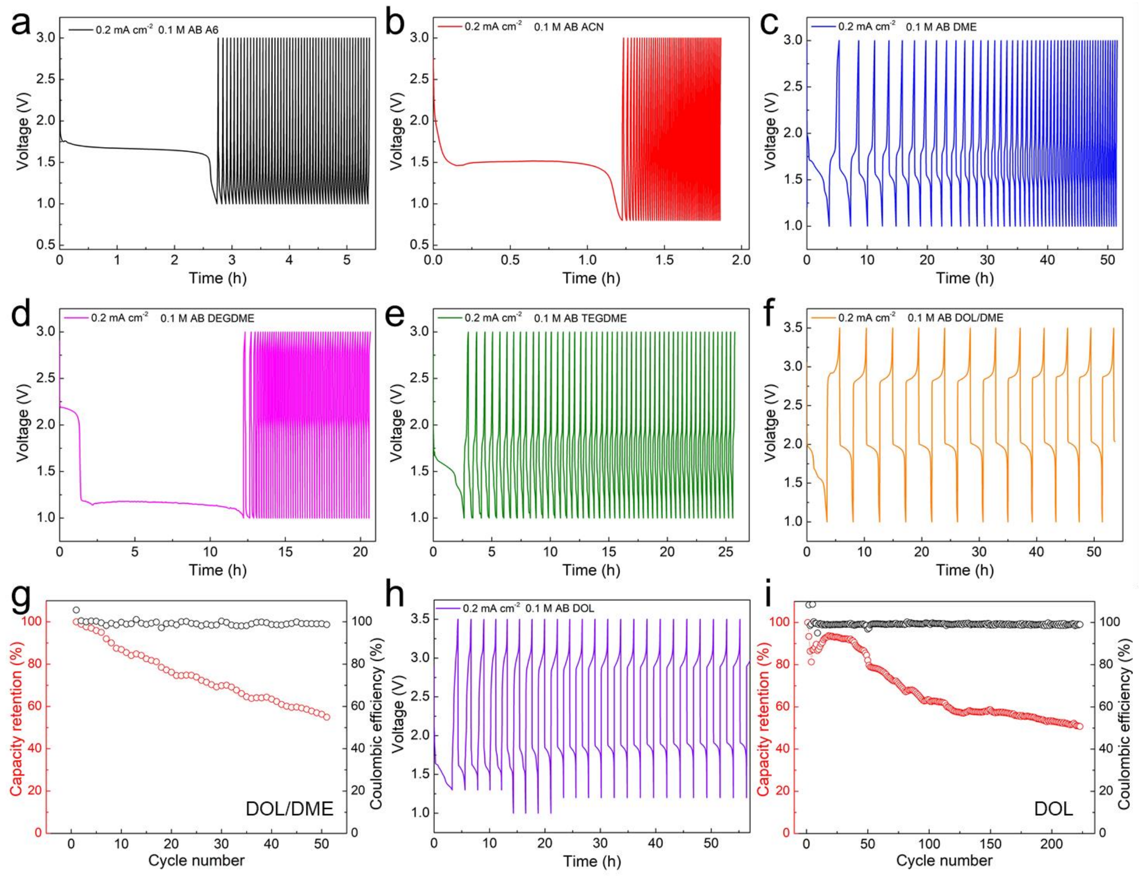

Supplementary Figure 11. Charge and discharge profiles of 0.1 M AB over time in different supporting electrolytes. a, Charge and discharge profiles of 0.1 M AB in A6 electrolytes at a current density of 0.2 mA cm^{-2}. **b**,**c**,**d**,**e**,**f**, Charge and discharge profiles of 0.1 M AB in ACN (**b**), DME (**c**), DEGDME (**d**), TEGDME (**e**) or DOL/DME (**f**) electrolytes with 0.5 M LITFSI at a current density of 0.2 mA cm^{-2}. **g**, Corresponding cycling capacity and coulombic efficiency of 0.1 M AB in DOL/DME supporting electrolytes at the current density of 0.2 mA cm^{-2}. **h**,**i**, Charge and discharge profiles of 0.1 M AB in DOL electrolytes with 0.5 M LITFSI at a current density of 0.2 mA cm^{-2} (**h**) and corresponding cycling capacity with coulombic efficiency (**i**).

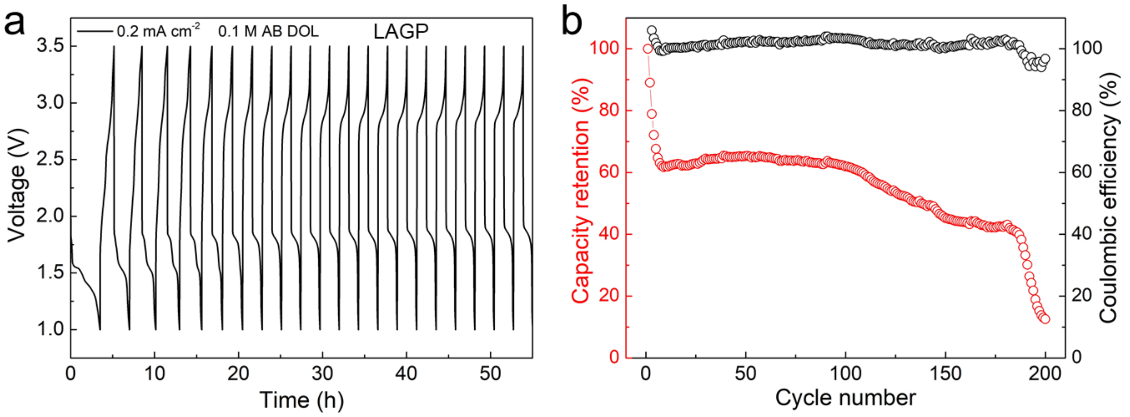

Supplementary Figure 12. a,**b**, Charge and discharge profiles of 0.1 M AB in 0.5 M LiTFSI DOL electrolytes using the LAGP separator at a current density of 0.2 mA cm^{-2} (**a**) and corresponding cycling capacity with coulombic efficiency (**b**).

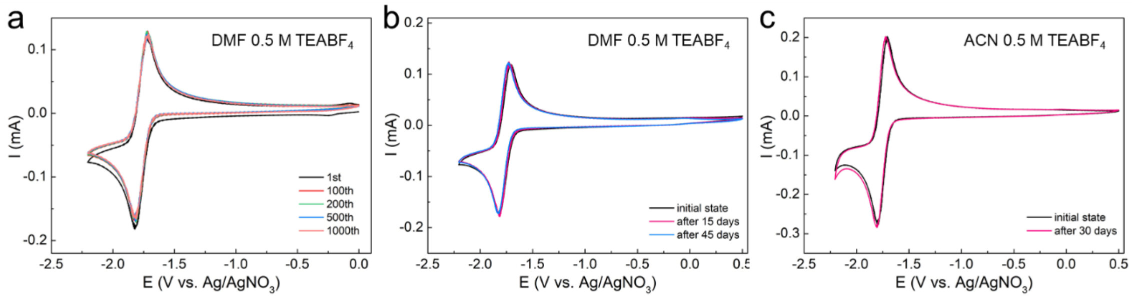

Supplementary Figure 13. a, CV curves of 10 mM AB in 0.5 M $TEABF_4$ DMF electrolytes at different specific cycles at a scan rate of 100 mV s^{-1}. **b**,**c**, CV curves of 10 mM AB in 0.5 M $TEABF_4$ DMF (**a**) or 0.5 M $TEABF_4$ ACN (**b**) electrolytes, respectively, after standing in glove box for different days at a scan rate of 100 mV s^{-1}.

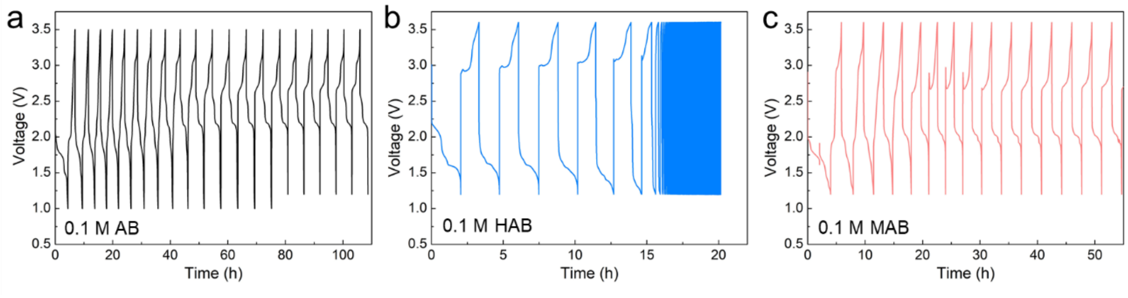

Supplementary Figure 14. a,**b**,**c**, Charge and discharge profiles of 0.1 M AB (**a**), 0.1 M HAB (**b**) and 0.1 M MAB (**c**) in DMF electrolytes with 0.5 M LiTFSI at a current density of 0.2 mA cm^{-2}.

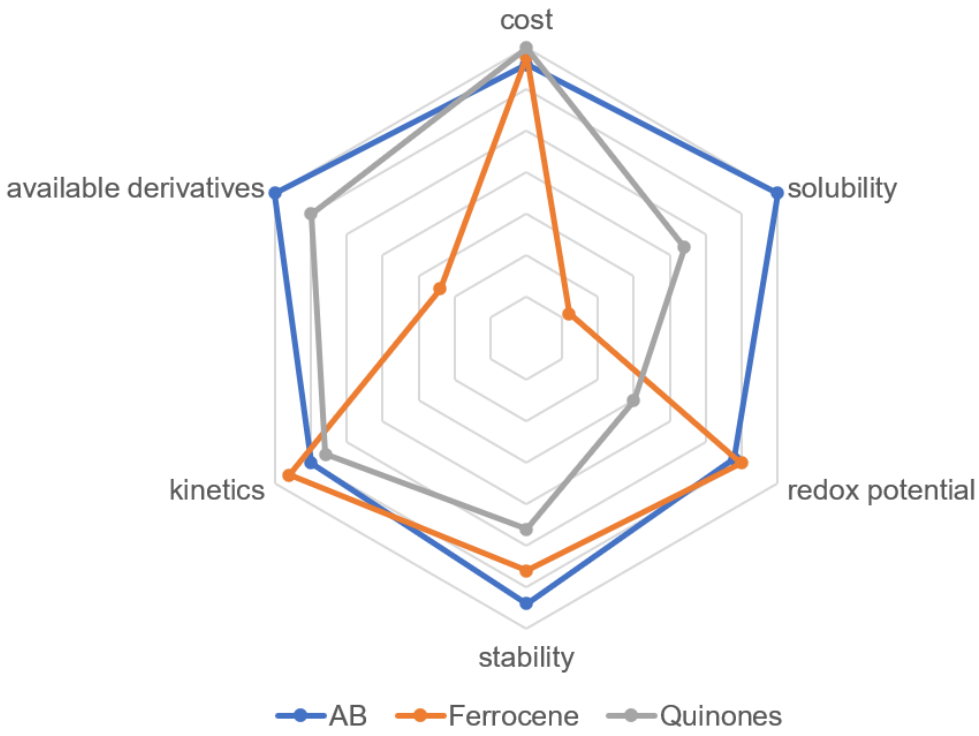

Supplementary Figure 15. Itemized comparison of AB with existing redox-active molecules for nonaqueous redox flow batteries.

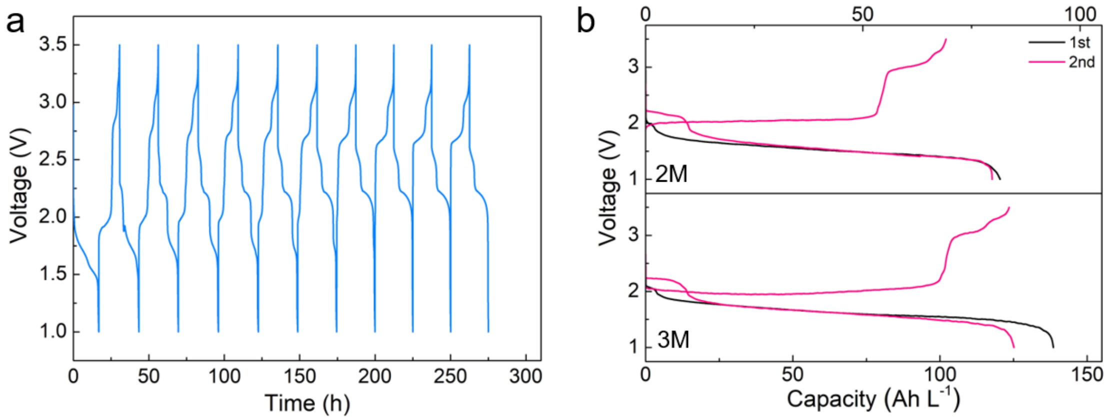

Supplementary Figure 16. a, Charge and discharge profiles of AB electrolyte (1 M) over time at the current density of 0.2 mA cm^{-2}. **b**, Charge and discharge profiles for 2 M or 3 M AB electrolytes at the current density of 0.2 mA cm^{-2}.

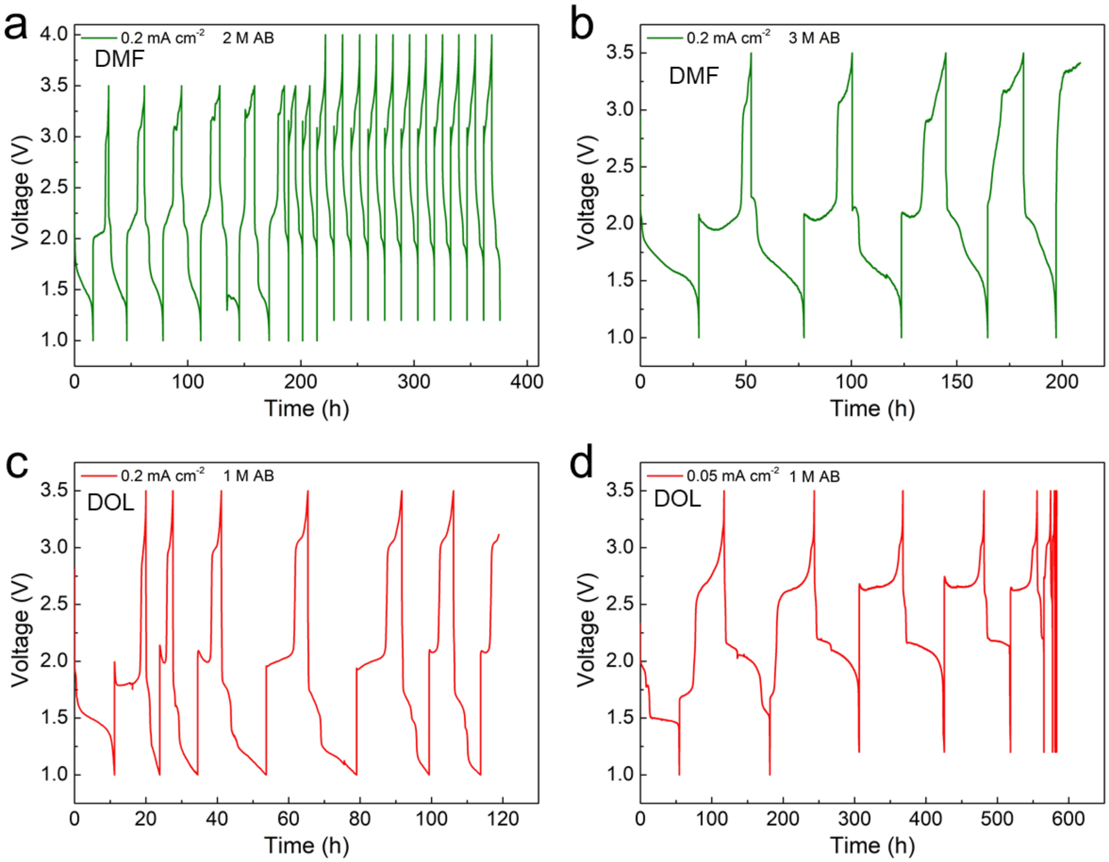

Supplementary Figure 17. a,**b**, Charge and discharge profiles of 2 M AB (**a**) or 3 M AB (**b**) electrolytes over time at the current density of 0.2 mA cm^{-2}. **c**,**d**, Charge and discharge profiles for 1 M AB in 1M LiTFSI DOL electrolytes at the current densities of 0.2 (**c**) and 0.05 mA cm^{-2} (**d**), respectively.

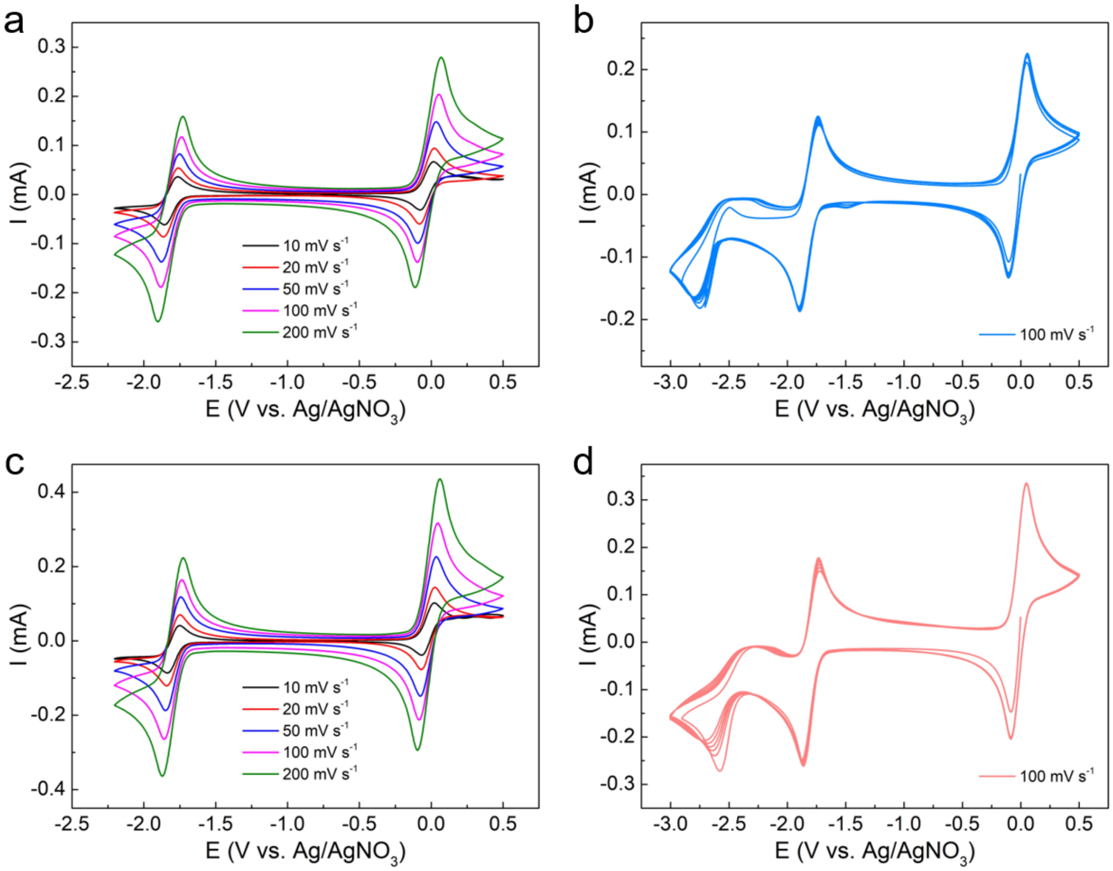

Supplementary Figure 18. **a**, CV curves of 10 mM AB and 10 mM Fc in 0.25 M $TBABF_4$ DMF electrolytes at various scan rates. **b**, CV curves of 10 mM AB and 10 mM Fc in 0.25 M $TBABF_4$ DMF electrolytes at a wide potential range (-3.0~0.5 V) at a scan rate of 100 mV s^{-1}. **c**, CV curves of 10 mM AB and 10 mM Fc in 0.25 M $TBABF_4$ ACN electrolytes at various scan rates. **d**, CV curves of 10 mM AB and 10 mM Fc in 0.25 M $TBABF_4$ ACN electrolytes at a wide potential range (-3.0~0.5 V) at a scan rate of 100 mV s^{-1}.

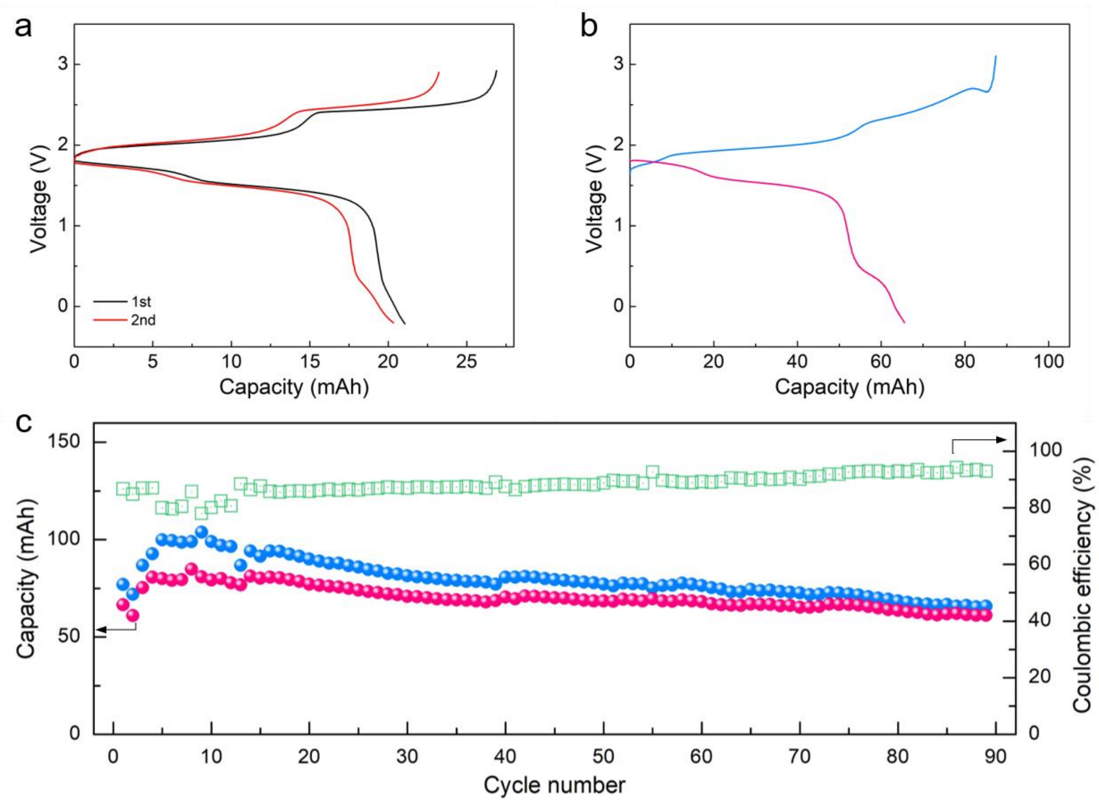

Supplementary Figure 19. **a**, Charge and discharge profiles of 0.1 M AB and Fc mixed electrolytes at the current density of 25 mA cm^{-2}. **b**, The initial voltage profile for 0.4 M AB and Fc mixed electrolytes at the current densities of 25 mA cm^{-2}. **c**, Cycling capacity and coulombic efficiency of the 0.4 M AB/Fc flow cell using mixed electrolytes (5 ml) cycled at 25 mA cm^{-2}.

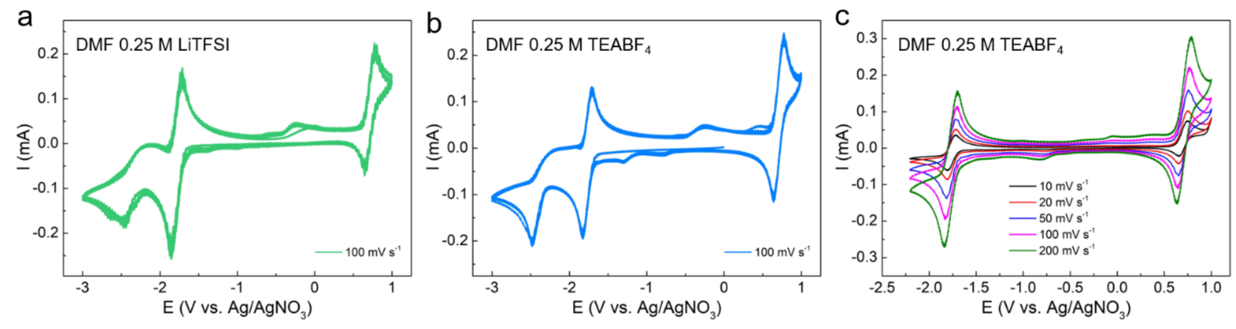

Supplementary Figure 20. a, CV curves of 10 mM AB and 10 mM DBMMB in 0.25 M LiTFSI DMF electrolytes at a scan rate of 100 mV s^{-1}. **b**, CV curves of 10 mM AB and 10 mM DBMMB in 0.25 M $TEABF_4$ DMF electrolytes at a scan rate of 100 mV s^{-1}. **c**, CV curves of 10 mM AB and 10 mM DBMMB in 0.25 M $TEABF_4$ DMF electrolytes at various scan rates.

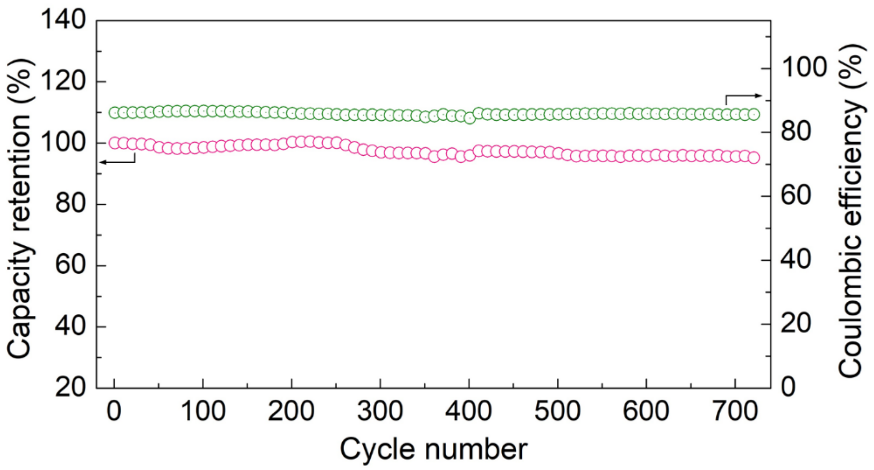

Supplementary Figure 21. Cycling capacity and coulombic efficiency of the 0.1 M AB/Fc cell using mixed electrolytes cycled at 10 mA cm^{-2}.

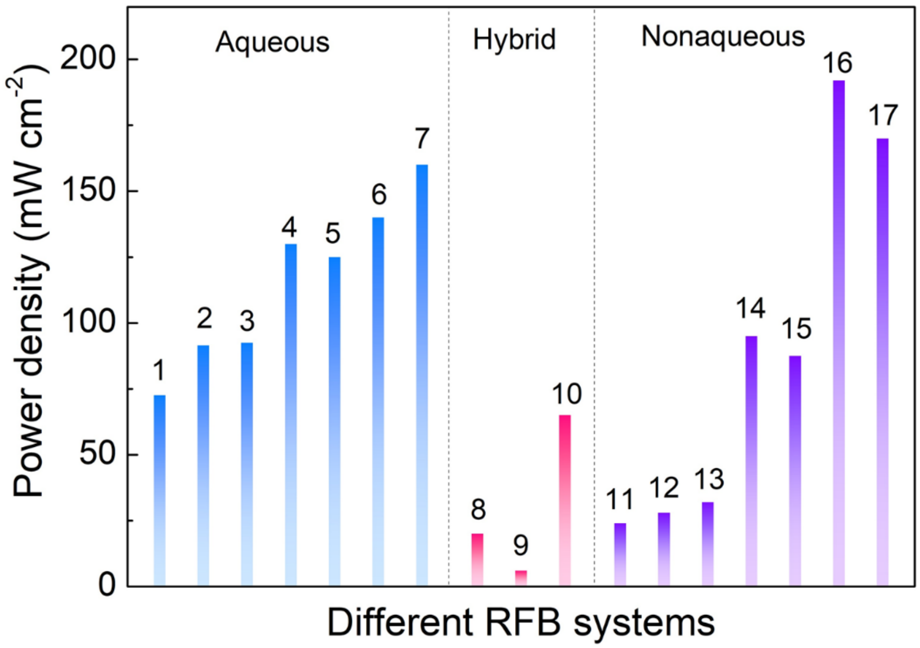

Supplementary Figure 22. Summarized power densities of various aqueous (1-7), hybrid (8-10) and nonaqueous (11-17) RFB systems. Note: The number 17 represents this work. Some values are extracted from the voltage profiles. (Reference for 1-7[1-7]; Reference for 8-10[8-10]; Reference for 11-16[11-16])

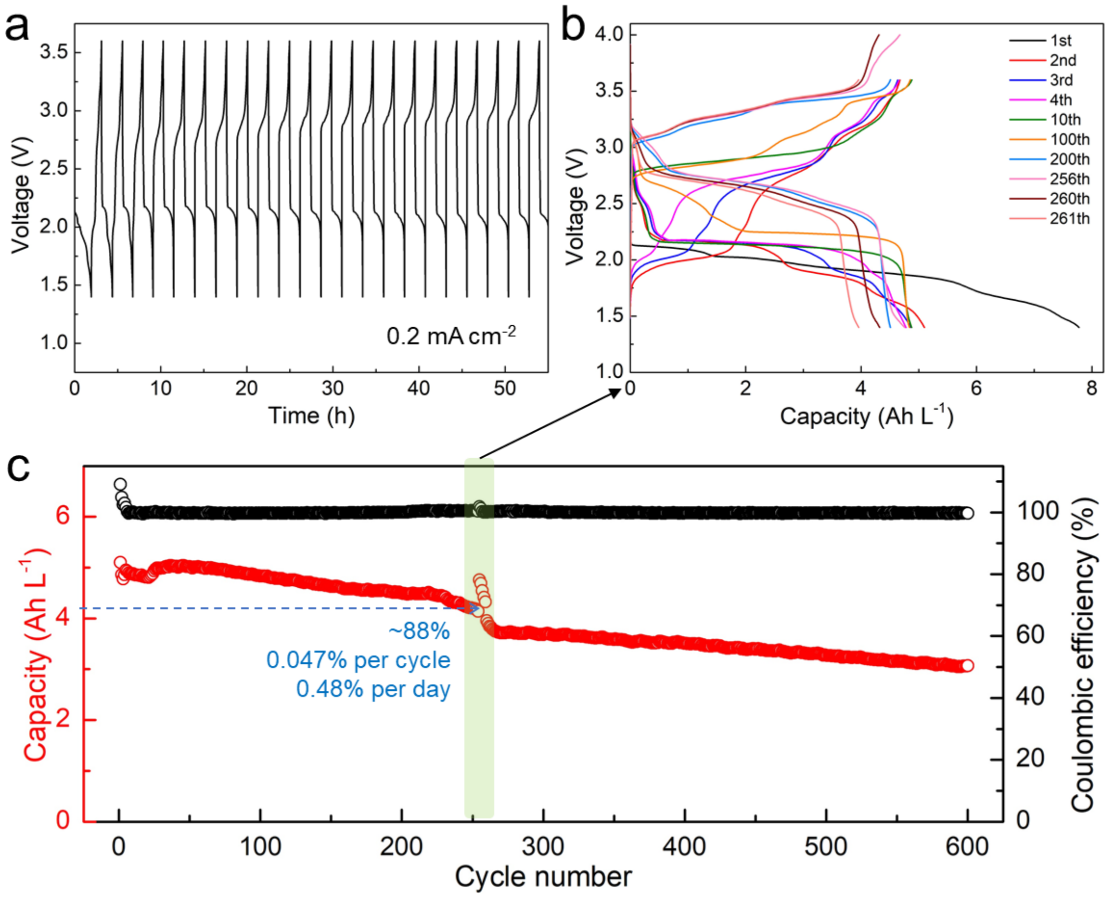

Supplementary Figure 23. a, Charge and discharge profiles of 0.1 M AB over time in DMF electrolytes with 0.4 M LiTFSI and 0.4 M $TEABF_4$ mixed supporting salts at a current density of 0.2 mA cm^{-2}. **b**, Representative voltage profiles versus capacity selected during the cycling test. **c**, Corresponding cycling capacity with coulombic efficiency at the current density of 0.2 mA cm^{-2}.

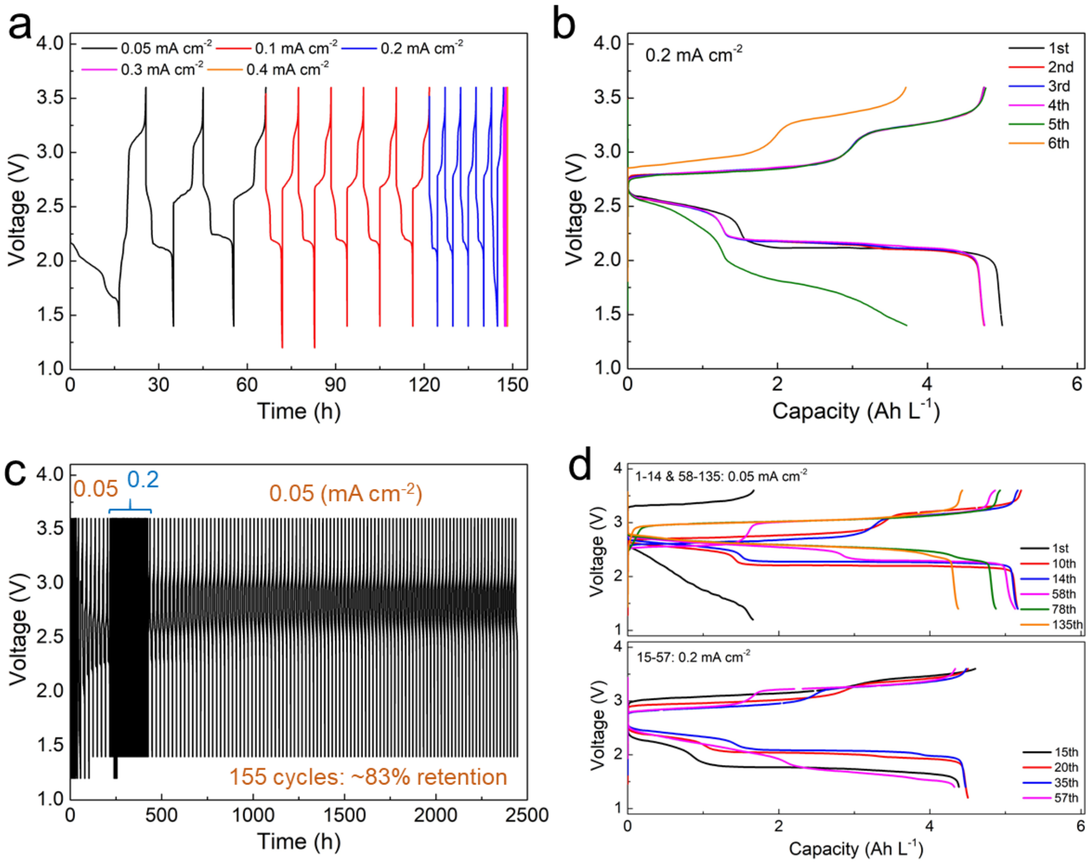

Supplementary Figure 24. Observation of stability of LATP membrane during the electrochemical test. a,**b**, Continuous charging and discharging of 0.1 M AB in DMF electrolytes with 0.1 M LiTFSI and 0.4 M $TEABF_4$ mixed supporting salts using LATP separators at various current densities (**a**) and following charging and discharging at a current density of 0.2 mA cm^{-2} (**b**). **c**,**d**, Charge and discharge profiles of 0.1 M AB in the electrochemical retest after (a) and (b) at the current densities of 0.05 and 0.2 mA cm^{-2} (**c**) and representative voltage profiles selected, considering the effect of current density (**d**).

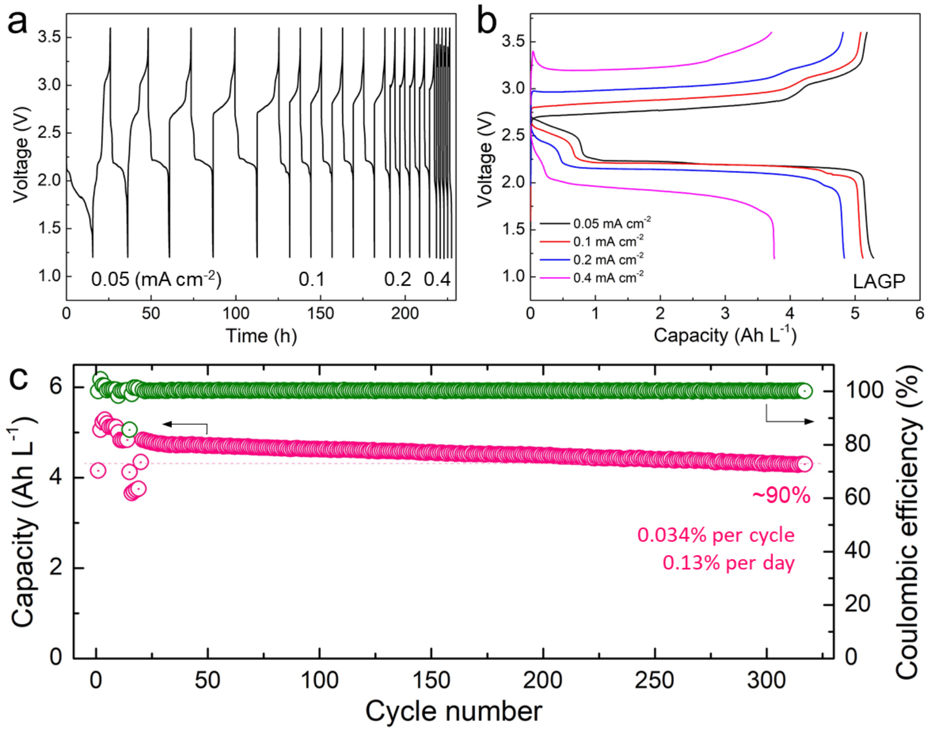

Supplementary Figure 25. a,**b**, Charge and discharge profiles of 0.1 M AB in 0.5 M LiTFSI DMF electrolyte at various current densities for rate capability test using the LAGP separator (**a**) and representative voltage profile versus capacity (**b**). **c**, Corresponding cycling capacity and coulombic efficiency at various current densities (0.05, 0.1, 0.2, 0.4 and 0.2 mA cm^{-2}).

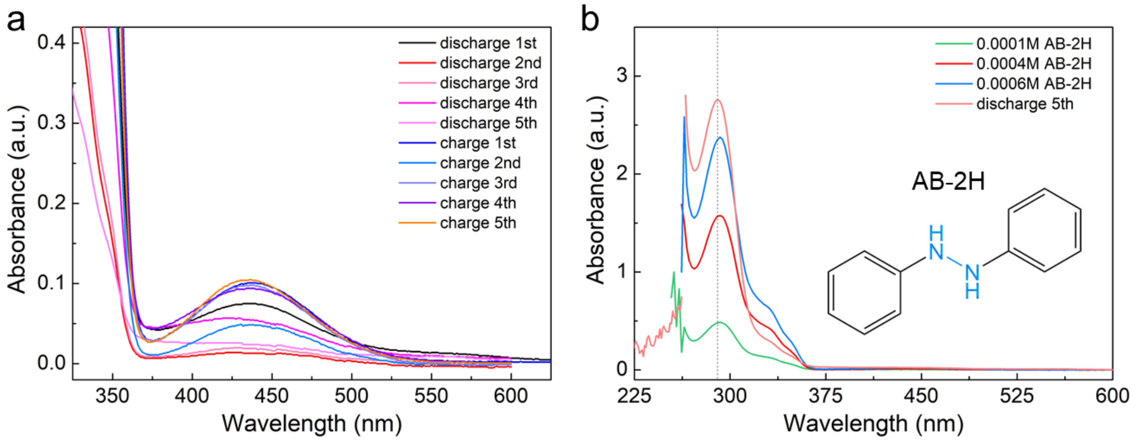

Supplementary Figure 26. a, UV-vis spectra of AB in the specific region of azo group (N=N) at different charging and discharging states. **b**, UV-vis spectra of hydrazobenzene (AB-2H) in DMF solvents.

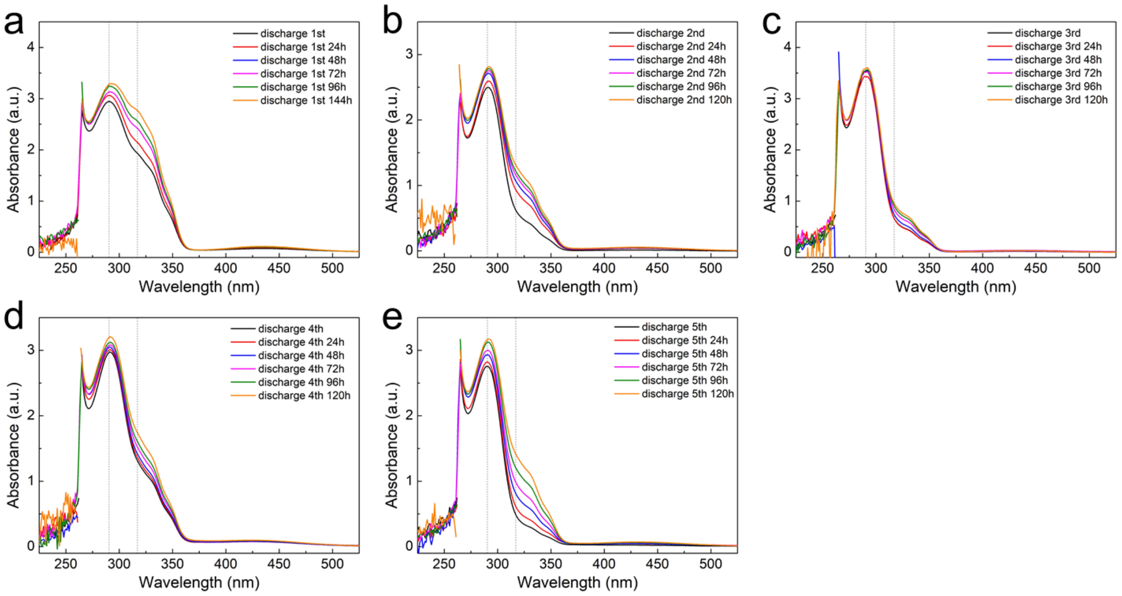

Supplementary Figure 27. UV-vis spectra of AB after discharging. **a**,**b**,**c**,**d**,**e**, UV-vis spectra of 0.1 M AB electrolytes with the change of time after the first discharging (**a**), second discharging (**b**), third discharging (**c**), fourth discharging (**d**) or fifth discharging (**e**), respectively.

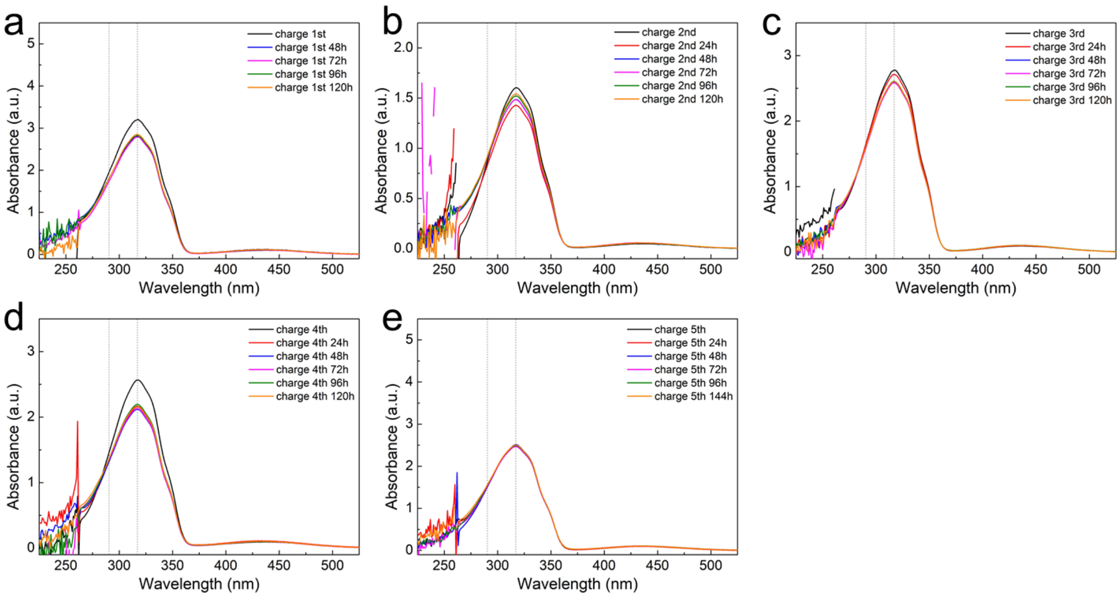

Supplementary Figure 28. UV-vis spectra of AB after charging. **a**,**b**,**c**,**d**,**e**, UV-vis spectra of 0.1 M AB electrolytes with the change of time after the first charging (**a**), second charging (**b**), third charging (**c**), fourth charging (**d**) or fifth charging (**e**), respectively.

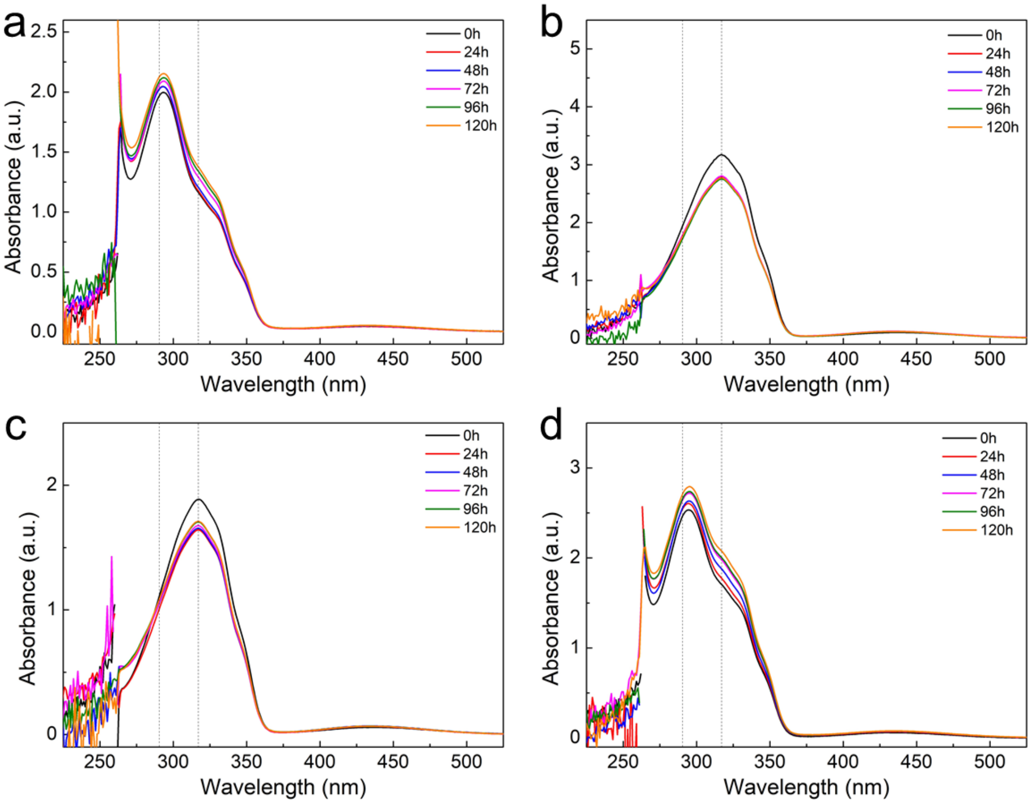

Supplementary Figure 29. UV-vis spectra of AB after the long cycling. **a**, UV-vis spectra over time of 0.1 M AB electrolytes cycled at 0.2 mA cm^{-2} for 300 cycles with a discharging state. **b**, UV-vis spectra over time of 0.1 M AB electrolytes at the charging state after cycling for 3000 cycles at 0.4 mA cm^{-2}. **c**, UV-vis spectra over time of 0.1 M AB electrolytes (0.4 M LiTFSI + 0.4 M $TEABF_4$) at the charging state after cycling for 600 cycles at 0.2 mA cm^{-2}. **d**, UV-vis spectra over time of 1 M AB electrolytes after 100 cycles at 0.2 mA cm^{-2} at the discharging state.

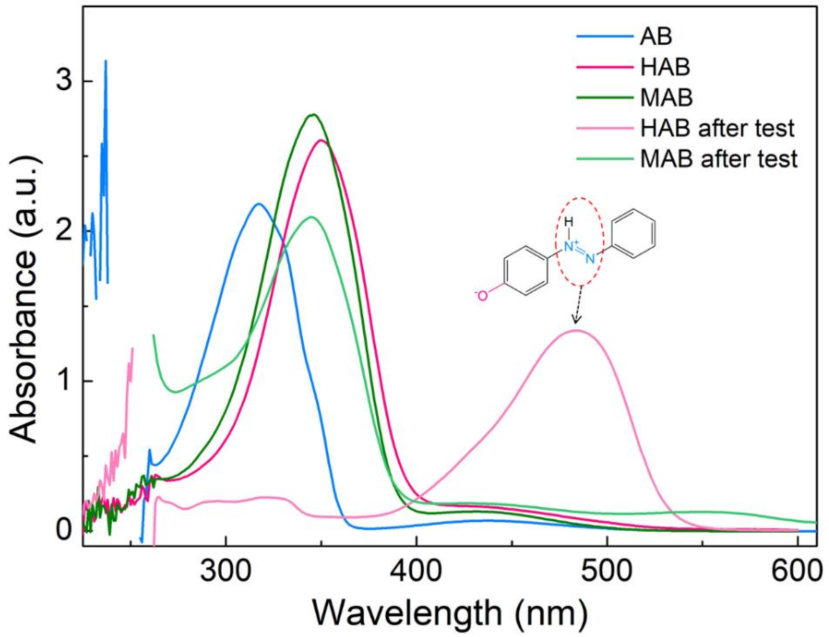

Supplementary Figure 30. UV-vis spectra of 0.1 M HAB and MAB electrolytes before and after battery test (charging state), respectively. The change of UV-vis spectrum of HAB after test should be ascribed to the protonation of azo group[17].

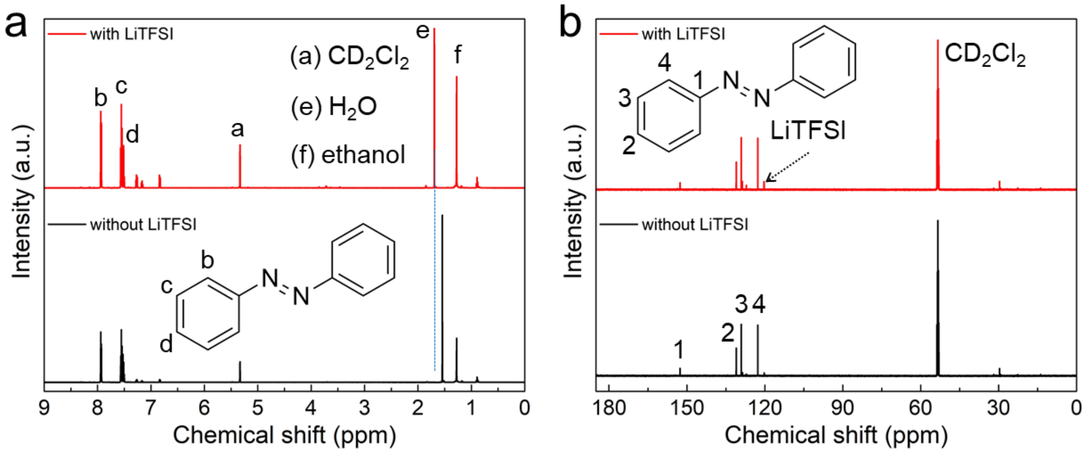

Supplementary Figure 31. a,**b**, ^{1}H NMR (**a**) and ^{13}C NMR (**b**) spectra for AB with or without LiTFSI salts using CD_2Cl_2 as the solvent. Due to the possible contamination from the cleaning liquids for NMR tubes (deionized water and ethanol), their signals were also detected.

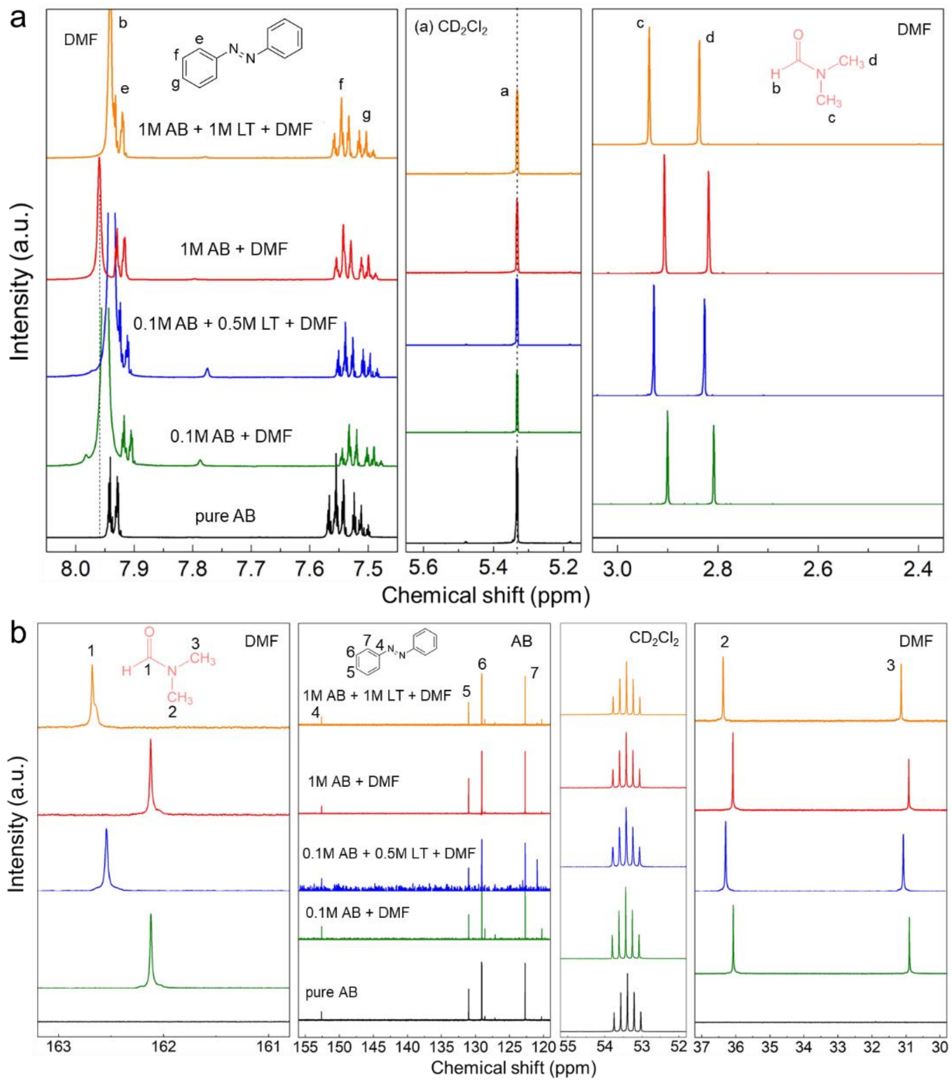

Supplementary Figure 32. a,**b**, ^{1}H NMR (**a**) and ^{13}C NMR (**b**) spectra for AB and AB electrolytes at a concentration of 0.1 M and 1 M without or with LiTFSI salts using CD_2Cl_2 as the solvent.

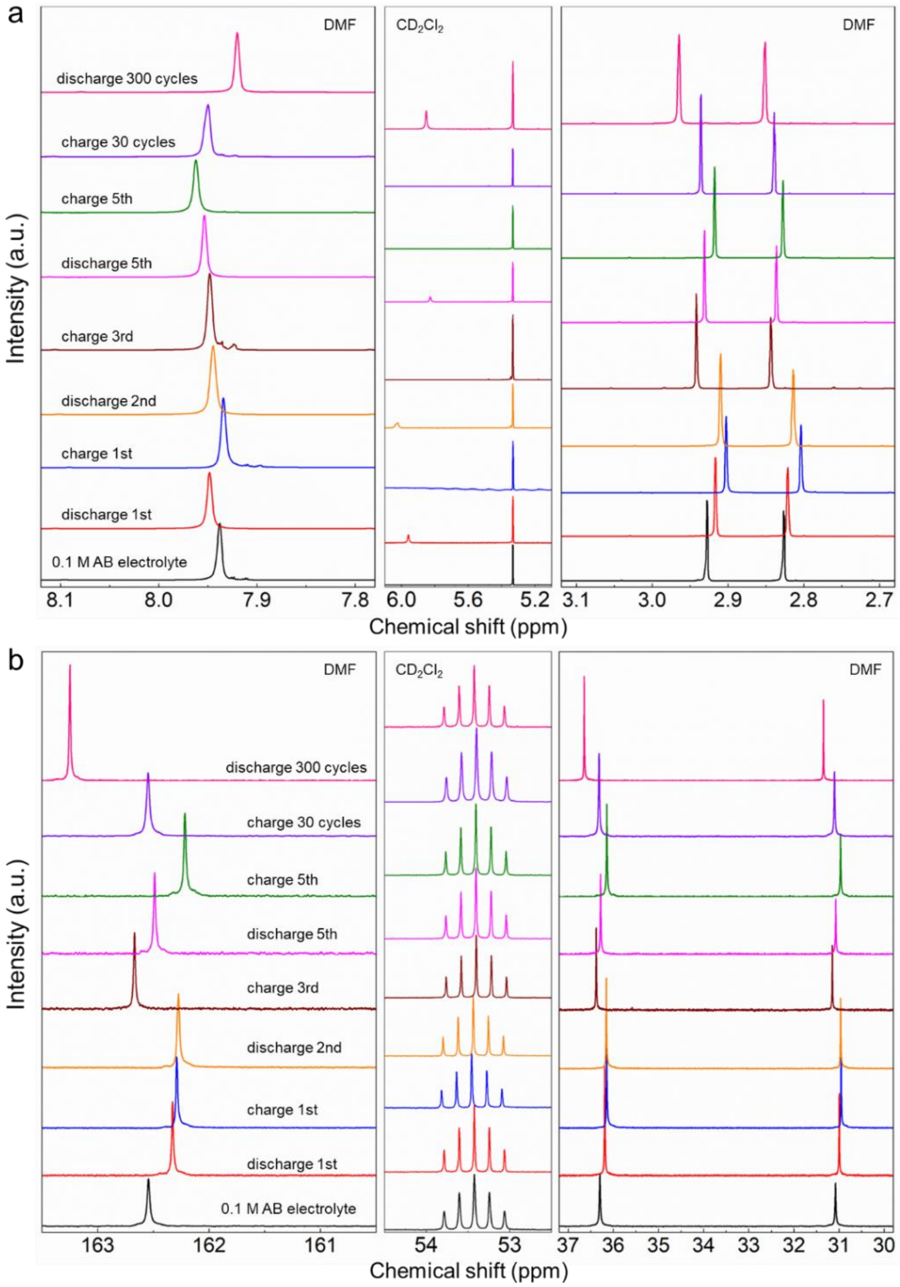

Supplementary Figure 33. a,**b**, ^{1}H NMR (**a**) and ^{13}C NMR (**b**) spectra of 0.1 M AB electrolytes recorded at various selected charge and discharge cycles or after cycling.

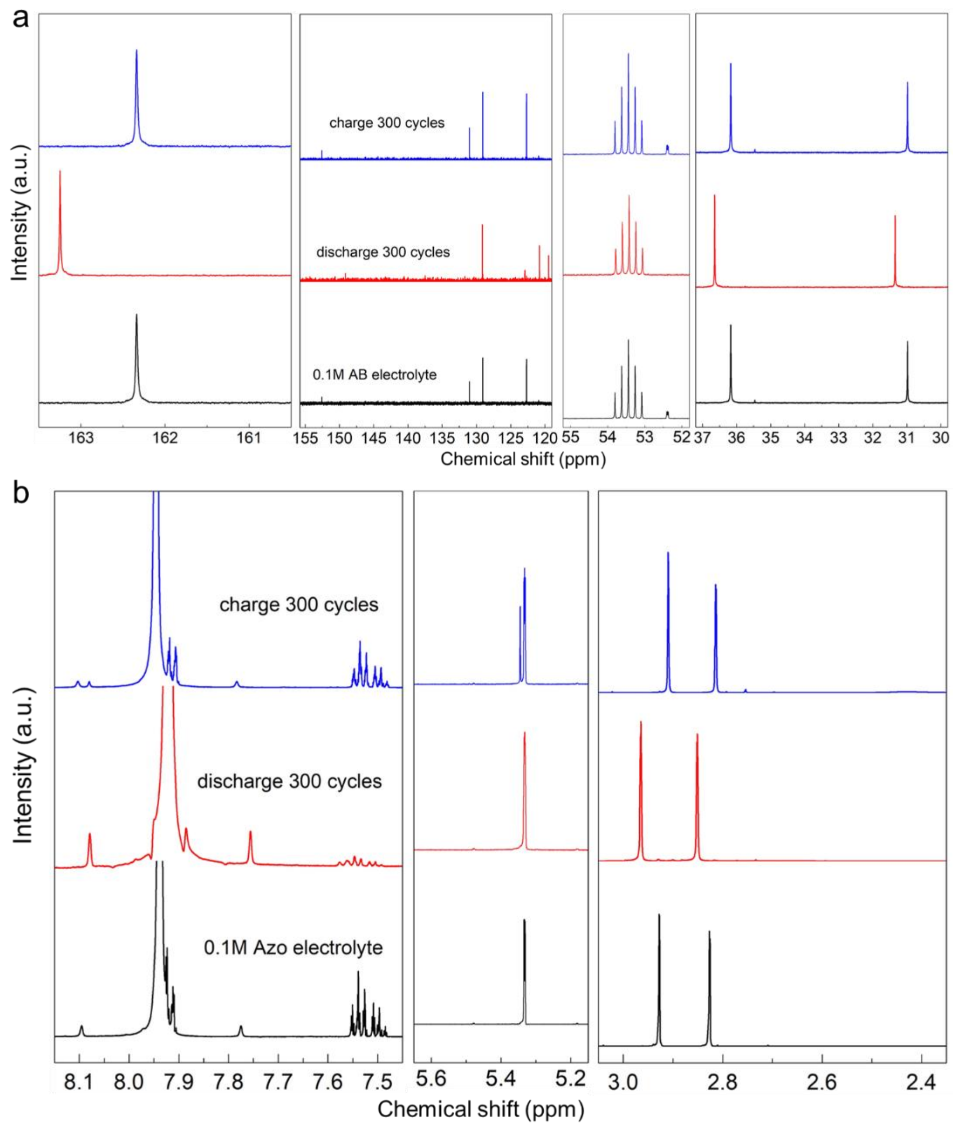

Supplementary Figure 34. a,**b**, ^{13}C NMR (**a**) and ^{1}H NMR (**b**) spectra for 0.1 M AB electrolytes after discharging and charging for a long time.

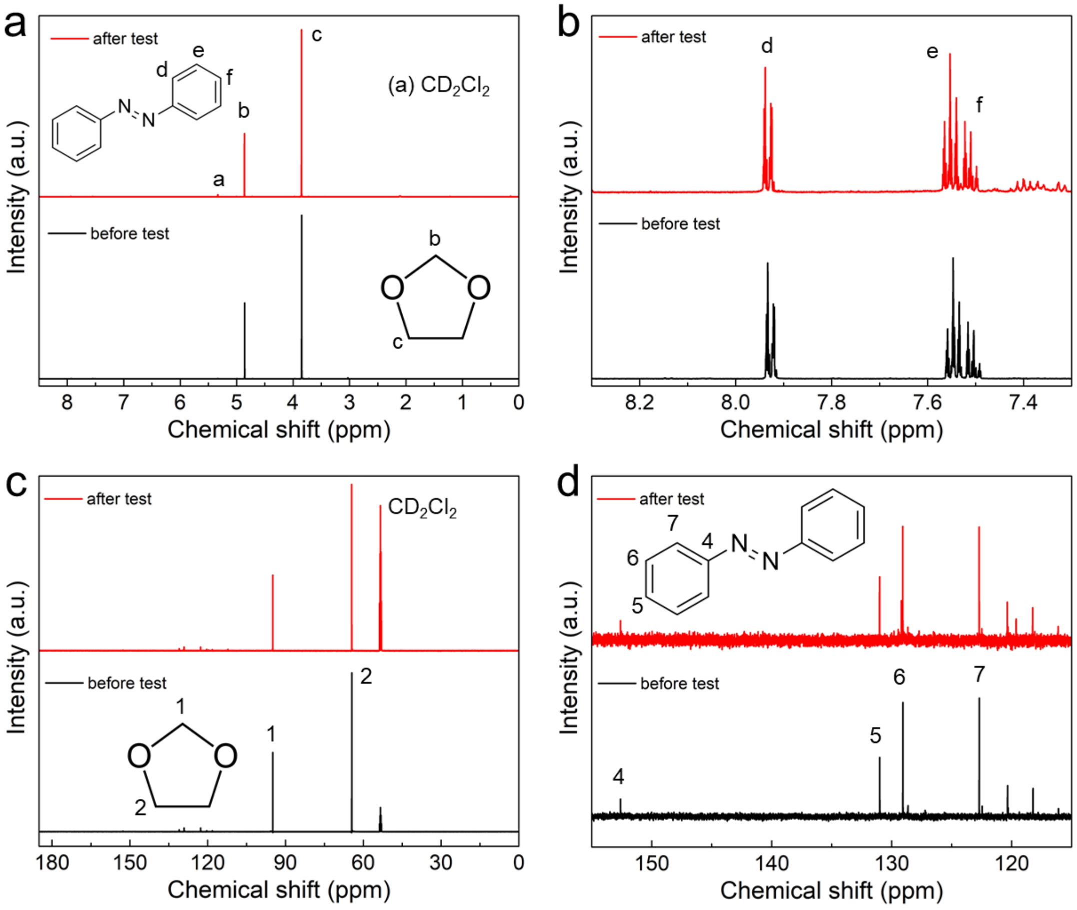

Supplementary Figure 35. **a**,**b**, ^{1}H NMR spectra of 0.1 M AB in 0.5 M LiTFSI DOL electrolytes before and after test (**a**) and corresponding peaks for AB molecule (**b**). **c**,**d**, ^{13}C NMR spectra of 0.1 M AB in 0.5 M LiTFSI DOL electrolytes before and after test (**c**) and corresponding peaks for AB molecule (**d**).

Supplementary Table 1. Kinetic parameters of redox species in aqueous and nonaqueous electrolytes.

Active materials	Measurement	Electrolytes	Diffusion coefficient / $cm^2\ s^{-1}$	Kinetic rate constant / $cm\ s^{-1}$
VO_2^+/VO^{2+} [18]	CV	1M H_2SO_4 aqueous	2.8×10^{-6}	6.8×10^{-5}
V^{3+}/V^{2+} [18]	CV	1M H_2SO_4 aqueous	2.4×10^{-6}	5.3×10^{-4}
MB[19]	LSV	3M H_2SO_4 aqueous	2.05×10^{-6}	0.32
$(Me)(NPr)V]Cl_3$ [4]	LSV	0.5M NaCl aqueous	5.4×10^{-6}	0.364
FMN-Na[20]	LSV	1M KOH aqueous	1.3×10^{-6}	5.3×10^{-3}
DMeOEPT[21]	CV	1M LiTFSI PC	1.2×10^{-6}	----
Ferrocene[22]	LSV	1M $LiPF_6$ DMF	8.9×10^{-6}	1.4×10^{-2}
BzNSN[23]	LSV	1M LiTFSI ACN	1.7×10^{-5}	9.0×10^{-3}
DBMMB[23]	LSV	1M LiTFSI ACN	7.7×10^{-6}	1.0×10^{-2}
MePh[15]	LSV	1M LiTFSI DME	8.38×10^{-6}	2.46×10^{-3}
DBMMB[15]	LSV	1M LiTFSI DME	5.77×10^{-6}	1.35×10^{-2}
AB (this work)	LSV	0.5M $TEABF_4$ DMF	7.75×10^{-6}	4.53×10^{-3}
AB (this work)	LSV	0.5M $TEABF_4$ ACN	2.85×10^{-5}	1.34×10^{-2}

Supplementary Table 2. Performance parameters of reported aqueous and nonaqueous RFBs.

Battery type	Concentration / M	Capacity / Ah L^{-1}	Energy density / Wh L^{-1}	Capacity retention / % (cycle no./time)	Capacity loss rate / % per cycle/per day
Aqueous redox flow batteries					
ACA/Fe[24]	0.5	20	18	91 (400/106h)	0.023/2.04
FMN/Fe[20]	0.06	2.6	2.8	>90 (200/124h)	
FMN/Fe[20]	0.24	10	9.6	99 (100/60h)	0.01/0.4
DHPS/Fe[25]	0.1	5	6.7	88 (1500/318h)	0.0079/0.93
DHPS/Fe[25]	1.4	67	94	90 (500/340h)	0.0195/0.68
V/MB[19]	0.1	5	3.5	81 (900/140h)	0.021/3.26
V/MB[19]	1.2	59	41	96 (160/300h)	0.025/0.52
MV/FcNCl[26]	0.5	11.8	7	91 (700/330h)	0.013/0.65
MV/4-HO-TEMPO[27]	0.5	9.6	6	89 (100/10h)	0.11/26.4
$(SPr)_2V/Fe$[1]	0.5	10.5	7.4	88 (300/110h)	0.04/2.62
Vi/TMAP-TEMPO[28]	0.1	2.4	2.3	94 (1000/220h)	0.006/0.62
Vi/TMAP-TEMPO[28]	0.5	11.8	10	95 (200/81h)	0.025/0.65
Nonaqueous redox flow batteries					
Li/TEMPO[29]	0.1	2.5	7.4	76 (100/30h)	0.24/19.2
Li/NQ[9]	0.1	2.1	5.3	98 (100/--)	0.02/--
Li/DMFc[30]	0.05	1.3	4	79 (1000/45h)	0.021/11.2
Li/Fc1N112TFSI[31]	0.1	--	--	95 (100/90h)	0.05/1.33
Li/Fc1N112TFSI[31]	0.8	17	50	83 (18/--)	0.95/--
Li/BenPh[32]	0.2	4.5	8	90 (50/--)	0.2/--
FL/DBMMB[33]	0.5	11.6	22	20 (100/--)	0.8/--
MePh/DBMMB[15]	0.3	6.2	14.3	88 (50/9h)	0.24/32
BzNSN/DBMMB[23]	0.1	2.1	4	83 (160/14h)	0.11/29.1
PTIO/PTIO[34]	0.1	2	3.4	50 (35/3h)	1.4/400
PTIO/PTIO[34]	0.5	10	17.3	40 (15/6h)	4/240
This work	0.1	4.6	10	85 (300/1450h)	0.05/0.24
This work	0.1	4.4	10	93 (650/1550h)	0.011/0.11
This work	0.1	3.2	7	75 (3000/1366h)	0.0083/0.44
This work	1	46	101	85 (100/2325h)	0.15/0.16

(All the capacity and energy density calculation are based on single electrolyte)

Supplementary Table 3. Comparison of Testing conditions and results of reported nonaqueous RFBs.

Battery type / active area	Anode / Cathode	Mode / Separator	Electrolyte	Current density	Capacity/ cycles/retention
Cylinder / --	1M Nap / 1M TEMPO	Static/BASE	0.2ml 1M $NaPF_6$ DME	5 mA	4 Ah L^{-1}/45/62%[35]
Coin cell / 1 cm^2	Li / 0.05M DMFc	Static/Celgard	10ul 1M $LiClO_4$ EC/DEC	0.6 mA cm^{-2}	1.3Ah L^{-1}/1000/79%[30]
Cylinder / 0.5 cm^2	Li / 0.1M NQ	Static/LATP	0.5M LiTFSI DMA	0.05 C	2.1 Ah L^{-1}/100/95%[9]
Cylinder / 0.5 cm^2	Li / 0.2M BenPh	Static/LAGP	--	0.2 mA cm^{-2}	4.5 Ah L^{-1}/50/90%[32]
Cylinder / 0.5 cm^2	Li / 0.1M Fc	Static/LATP	1M $LiPF_6$ DMF	0.2 C	--/250/90%[22]
Cylinder / --	0.1M BuPh-DMFc	--/porous	1M $TEABF_4$ ACN	60 mA cm^{-2}	~1 Ah L^{-1}/500/72%[36]
MF-1056 / --	Li / 1mM DMeOEPT	Static/--	30ml 1M LiTFSI PC	0.804 mA	0.05 Ah L^{-1}/50/90%[21]
Stack / 4 cm^2	0.2M FL / 0.1M BMEPZ	Flow / porous	13ml 0.5M LiTFSI ACN	20 mA cm^{-2}	3.9 Ah L^{-1}/100/90%[37]
Stack / 10 cm^2	0.1M PTIO / 0.1M PTIO	Flow / porous	4ml 1M $TBAPF_6$ ACN	20 mA cm^{-2}	1.7 Ah L^{-1}/35/50%[34]
Stack / 20 cm^2	0.5M FL / 0.5M DBMMB	Flow / porous	1M TEA-TFSI ACN	15 mA cm^{-2}	9.5 Ah L^{-1}/100/20%[33]
Stack / 40 cm^2	Li / 0.1M TEMPO	Flow / porous	13ml 1M $LiPF_6$ EC/PC/EMC	5 mA cm^{-2}	2.5 Ah L^{-1}/100/76%[29]
Stack / 20 cm^2	0.3M MePh / DBMMB	Flow / porous	10ml 1M LiTFSI DME	35 mA cm^{-2}	6 Ah L^{-1}/50/90%[15]
Stack / 5 cm^2	0.1M BzNSN / DBMMB	Flow / porous	4ml 1M LiTFSI ACN	40 mA cm^{-2}	2.2 Ah L^{-1}/160/83%[23]
Stack / 4 cm^2	1M BuPh-DMFc	Flow / porous	1M $TEABF_4$ ACN	60 mA cm^{-2}	10 Ah L^{-1}/20/--[36]
Cylinder / 0.5 cm^2	Li / 0.1M AB	Static/LATP	0.5M LiTFSI DMF	0.2 mA cm^{-2}	4.4 Ah L^{-1}/1000/86%
Cylinder / 0.5 cm^2	Li / 1M AB	Static/LATP	1M LiTFSI DMF	0.2 mA cm^{-2}	46 Ah L^{-1}/100/85%
Cylinder / --	0.1M AB / Fc	--/porous	1M LiTFSI DMF	10 mA cm^{-2}	4.5 Ah L^{-1}/700/95%
Stack / 4 cm^2	0.1M AB / Fc	Flow / porous	5ml 0.5M LiTFSI DMF	25 mA cm^{-2}	4.2 Ah L^{-1}/450/86%
Stack / 4 cm^2	0.4M AB / Fc	Flow / porous	5ml 1M LiTFSI DMF	25 mA cm^{-2}	17 Ah L^{-1}/90/76%

(This table is primarily focused on comparing the battery test methods applied in reported works for nonaqueous redox flow batteries. And generally, the mixed electrolytes are used for the battery test in the flow mode. The calculated capacity is based on the single electrolyte volume)

Supplementary References

1 Luo, J. *et al.* Unprecedented Capacity and Stability of Ammonium Ferrocyanide Catholyte in pH Neutral Aqueous Redox Flow Batteries. *Joule* **3**, 149-163 (2019).

2 Hu, B., Luo, J., Hu, M., Yuan, B. & Liu, T. L. A pH-Neutral, Metal-Free Aqueous Organic Redox Flow Battery Employing an Ammonium Anthraquinone Anolyte. *Angew. Chem. Int. Ed.* **131**, 16782-16789 (2019).

3 DeBruler, C., Hu, B., Moss, J., Luo, J. & Liu, T. L. A sulfonate-functionalized viologen enabling neutral cation exchange, aqueous organic redox flow batteries toward renewable energy storage. *ACS Energy Lett.* **3**, 663-668 (2018).

4 DeBruler, C. *et al.* Designer two-electron storage viologen anolyte materials for neutral aqueous organic redox flow batteries. *Chem* **3**, 961-978 (2017).

5 Hu, B., DeBruler, C., Rhodes, Z. & Liu, T. L. Long-cycling aqueous organic redox flow battery (AORFB) toward sustainable and safe energy storage. *J. Am. Chem. Soc.* **139**, 1207-1214 (2017).

6 Jin, S. *et al.* Near Neutral pH Redox Flow Battery with Low Permeability and Long-Lifetime Phosphonated Viologen Active Species. *Adv. Energy Mater.* **10**, 2000100 (2020).

7 Orita, A., Verde, M. G., Sakai, M. & Meng, Y. S. A biomimetic redox flow battery based on flavin mononucleotide. *Nat. Commun.* **7**, 1-8 (2016).

8 Zhang, L., Zhang, C., Ding, Y., Ramirez-Meyers, K. & Yu, G. A low-cost and high-energy hybrid iron-aluminum liquid battery achieved by deep eutectic solvents. *Joule* **1**, 623-633 (2017).

9 Ding, Y., Li, Y. & Yu, G. Exploring bio-inspired quinone-based organic redox flow batteries: a combined experimental and computational study. *Chem* **1**, 790-801 (2016).

10 Baclig, A. C. *et al.* High-voltage, room-temperature liquid metal flow battery enabled by Na-K| K-β ″-alumina stability. *Joule* **2**, 1287-1296 (2018).

11 Kwon, G. *et al.* Bio-inspired Molecular Redesign of a Multi-redox Catholyte for High-Energy Non-aqueous Organic Redox Flow Batteries. *Chem* **5**, 2642-2656 (2019).

12 Duan, W. *et al.* A symmetric organic-based nonaqueous redox flow battery and its state of charge diagnostics by FTIR. *J. Mater. Chem. A* **4**, 5448-5456 (2016).

13 Wei, X. *et al.* Radical compatibility with nonaqueous electrolytes and its impact on an all-organic redox flow battery. *Angew. Chem. Int. Ed.* **54**, 8684-8687 (2015).

14 Duan, W. *et al.* "Wine-Dark Sea" in an organic flow battery: storing negative charge in 2, 1, 3-benzothiadiazole radicals leads to improved cyclability. *ACS Energy Lett.* **2**, 1156-1161 (2017).

15 Wei, X. *et al.* A high-current, stable nonaqueous organic redox flow battery. *ACS Energy Lett.* **1**, 705-711 (2016).

16 Zhang, C. *et al.* Biredox Eutectic Electrolytes Derived from Organic Redox-Active Molecules: High-Energy Storage Systems. *Angew. Chem. Int. Ed.* **131**, 7119-7124 (2019).

17 Chandra, S. *et al.* Phosphoric Acid Loaded Azo (−N═N−) Based Covalent Organic Framework for Proton Conduction. *J. Am. Chem. Soc.* **136**, 6570-6573 (2014).

18 Yamamura, T., Watanabe, N., Yano, T. & Shiokawa, Y. Electron-Transfer Kinetics of Np^{3+}/Np^{4+}, NpO^{2+}/NpO_2^{2+}, V^{2+}/V^{3+}, and VO_2^{+}/VO^{2+} at Carbon Electrodes. *J. Electrochem. Soc.* **152**, A830 (2005).

19 Zhang, C. *et al.* Phenothiazine-Based Organic Catholyte for High-Capacity and Long-Life Aqueous Redox Flow Batteries. *Adv. Mater.* **31**, e1901052 (2019).

20 Orita, A., Verde, M. G., Sakai, M. & Meng, Y. S. A biomimetic redox flow battery based on flavin mononucleotide. *Nat. Commun.* **7**, 1-8 (2016).

21 Kowalski, J. A. *et al.* A stable two-electron-donating phenothiazine for application in nonaqueous redox flow batteries. *J. Mater. Chem. A* **5**, 24371-24379 (2017).

22 Zhao, Y. *et al.* Sustainable Electrical Energy Storage through the Ferrocene/Ferrocenium Redox Reaction in Aprotic Electrolyte. *Angew. Chem. Int. Ed.* **53**, 11036-11040 (2014).

23 Duan, W. *et al.* “Wine-Dark Sea” in an Organic Flow Battery: Storing Negative Charge in 2,1,3-Benzothiadiazole Radicals Leads to Improved Cyclability. *ACS Energy Lett.* **2**, 1156-1161 (2017).
24 Lin, K. *et al.* A redox-flow battery with an alloxazine-based organic electrolyte. *Nat. Energy* **1**, 16102 (2016).
25 Hollas, A. *et al.* A biomimetic high-capacity phenazine-based anolyte for aqueous organic redox flow batteries. *Nat. Energy* **3**, 508-514 (2018).
26 Hu, B., DeBruler, C., Rhodes, Z. & Liu, T. L. Long-Cycling Aqueous Organic Redox Flow Battery (AORFB) toward Sustainable and Safe Energy Storage. *J. Am. Chem. Soc.* **139**, 1207-1214 (2017).
27 Liu, T., Wei, X., Nie, Z., Sprenkle, V. & Wang, W. A Total Organic Aqueous Redox Flow Battery Employing a Low Cost and Sustainable Methyl Viologen Anolyte and 4-HO-TEMPO Catholyte. *Adv. Energy Mater.* **6**, 1501449 (2016).
28 Liu, Y. *et al.* A Long-Lifetime All-Organic Aqueous Flow Battery Utilizing TMAP-TEMPO Radical. *Chem* **5**, 1861-1870 (2019).
29 Wei, X. *et al.* TEMPO-based catholyte for high-energy density nonaqueous redox flow batteries. *Adv. Mater.* **26**, 7649-7653 (2014).
30 Cong, G., Zhou, Y., Li, Z. & Lu, Y.-C. A Highly Concentrated Catholyte Enabled by a Low-Melting-Point Ferrocene Derivative. *ACS Energy Lett.* **2**, 869-875 (2017).
31 Wei, X. *et al.* Towards High-Performance Nonaqueous Redox Flow Electrolyte Via Ionic Modification of Active Species. *Adv. Energy Mater.* **5**, 1400678 (2015).
32 Zhang, C. *et al.* Highly Concentrated Phthalimide-Based Anolytes for Organic Redox Flow Batteries with Enhanced Reversibility. *Chem* **4**, 2814-2825 (2018).
33 Wei, X. *et al.* Radical Compatibility with Nonaqueous Electrolytes and Its Impact on an All-Organic Redox Flow Battery. *Angew. Chem. Int. Ed.* **54**, 8684-8687 (2015).
34 Duan, W. *et al.* A symmetric organic-based nonaqueous redox flow battery and its state of charge diagnostics by FTIR. *J. Mater. Chem. A* **4**, 5448-5456 (2016).
35 Wang, G. *et al.* Exploring polycyclic aromatic hydrocarbons as an anolyte for nonaqueous redox flow batteries. *J. Mater. Chem. A* **6**, 13286-13293 (2018).
36 Zhang, C. *et al.* Biredox Eutectic Electrolytes Derived from Organic Redox-Active Molecules: High-Energy Storage Systems. *Angew. Chem. Int. Ed.* **58**, 7045-7050 (2019).
37 Kwon, G. *et al.* Bio-inspired Molecular Redesign of a Multi-redox Catholyte for High-Energy Non-aqueous Organic Redox Flow Batteries. *Chem* **5**, 2642-2656 (2019).
